# PG-Metrics: A chemometric-based approach for classifying bacterial peptidoglycan data sets and uncovering their subjacent chemical variability

**DOI:** 10.1371/journal.pone.0186197

**Published:** 2017-10-17

**Authors:** Keshav Kumar, Akbar Espaillat, Felipe Cava

**Affiliations:** Laboratory for Molecular Infection Medicine Sweden, Department of Molecular Biology, Umeå Centre for Microbial Research, Umeå University, Umeå, Sweden; Institut Pasteur Paris, FRANCE

## Abstract

Bacteria cells are protected from osmotic and environmental stresses by an exoskeleton-like polymeric structure called peptidoglycan (PG) or murein sacculus. This structure is fundamental for bacteria’s viability and thus, the mechanisms underlying cell wall assembly and how it is modulated serve as targets for many of our most successful antibiotics. Therefore, it is now more important than ever to understand the genetics and structural chemistry of the bacterial cell walls in order to find new and effective methods of blocking it for the treatment of disease. In the last decades, liquid chromatography and mass spectrometry have been demonstrated to provide the required resolution and sensitivity to characterize the fine chemical structure of PG. However, the large volume of data sets that can be produced by these instruments today are difficult to handle without a proper data analysis workflow. Here, we present PG-metrics, a chemometric based pipeline that allows fast and easy classification of bacteria according to their muropeptide chromatographic profiles and identification of the subjacent PG chemical variability between e.g. bacterial species, growth conditions and, mutant libraries. The pipeline is successfully validated here using PG samples from different bacterial species and mutants in cell wall proteins. The obtained results clearly demonstrated that PG-metrics pipeline is a valuable bioanalytical tool that can lead us to cell wall classification and biomarker discovery.

## Introduction

Bacterial cytoplasmic membrane is surrounded by a net-like polymeric structure named the peptidoglycan (PG) cell wall, which provides protection against environmental stresses, osmotic pressure and, defines bacterial shape. Peptidoglycan is a fundamental structure for the survival of most bacteria and thus, it is a major target of the β-lactams antibiotics. Peptidoglycan canonical composition is based on glycan strands of variable length composed of N-acetyl-glucosamine-β(1→4)-N-acetyl-muramic acid disaccharide repeats crosslinked by means of short peptide stems [1]. This composition, although highly conserved, is not unique and thus, certain bacterial species might exhibit distinct PG chemical structures which can even vary in response to environmental cues [2]. Far from being merely decorative, PG chemical variability is instrumental for bacteria to cope with a number of threats such as immune responses, predatory hydrolytic enzymes and antibiotics [3–5]. Moreover, specific PG structures are associated with the transition to diverse developmental stages, growth phases and cellular morphologies [6–8]. Therefore, a comprehensive analysis of the PG chemical structure in a large number of species and experimental conditions is fundamental for a better understanding of cell wall biology, in particular its role in signalling and environmental adaptation.

The most informative way of analysing bacteria’s PG fine chemical structure is by means of liquid chromatography combined with mass spectrometry (MS). The introduction of high-performance liquid chromatography (HPLC), and more recently its improved version the ultra-performance liquid chromatography (UPLC)-coupled to either UV-visible or MS detector, has provided accurate and quantitative information of the PG architecture. UPLC systems’ superior sensitivity provides better quality of chromatograms using less sample and to perform much shorter runs with comparable resolution. These technological advances have opened the door for high throughput PG analytical screenings, something completely unconceivable a few years ago [5, 9].

Similarly to any *omic*, one of the issues encountered when dealing with large data sets is the difficulties to make sense of this information. Peptidoglycan profiles are rather complex spectra composed of several tens of components (i.e. muropeptides), sometimes partially co-eluting and with high internal variability in number, type and relative abundance between species [2]. In addition to this biological variability, comparison of chromatograms acquired over a period of time suffers from technical issues. Often, two chromatograms obtained for the same sample on two different instruments show drifts in retention times, i.e. same information is stored at different time points for different spectra. These drifts are practically inevitable and technically introduced by (i) temperature fluctuations in the column, (ii) suboptimal mobile phase mixing, (iii) deterioration of the stationary phase or, (iv) the interaction between analytes [10, 11]. Moreover, the retention time drifts across the sample sets are irregular, meaning that chromatograms of different samples can have expansions and compressions of different segments. In conclusion, manual analysis of large PG data sets is laborious, time consuming and it has higher risks to generate misleading results as a consequence of imperfect alignments between samples. Therefore, an approach where the system is analysed using the integral response of the constituents is far more practical and feasible than manual expert analysis.

Chemometrics is a chemical discipline that uses advanced statistical methods for the efficient and fast analysis of large data sets generated by analytical instruments such as UPLC, nuclear magnetic resonance (NMR), infrared, UV-visible, fluorescence or Raman spectroscopy [12, 13]. Chemometrics techniques are generally used for sample classification and outlier identification. The classification approach can further be categorised as (i) un-supervised and (ii) supervised pattern recognition techniques [13]. The application of unsupervised pattern recognition techniques does not require *a priori* information about the group to which a sample belongs and the samples with similar characteristics cluster naturally. On the other hand, application of supervised pattern recognition technique requires *a priori* knowledge about the group to which a particular sample belongs. Often, such *a priori* information is not available and thus, it limits the application of supervised pattern recognition techniques. The most commonly used unsupervised pattern recognition technique is the principal component analysis (PCA) [12–15]. In essence, PCA simplifies and reduces the dimension of the data set by finding and projecting the original data set in a space spanned by fewer number of axes that are orthogonal to each other. With a few factors, PCA captures all the important information in the data sets. Theoretical aspects of PCA are well described and can be found elsewhere [12–15]. Importantly, PCA has been successfully used for the analysis of data sets obtained from different fields [16–23]. If PCA results are carefully analysed with appropriate mathematics and statistical parameters, it will allow to find: (i) natural clustering of the samples, (ii) the relation between the different groups in the study and, (iii) outlier samples [24].

**Fig 1:**
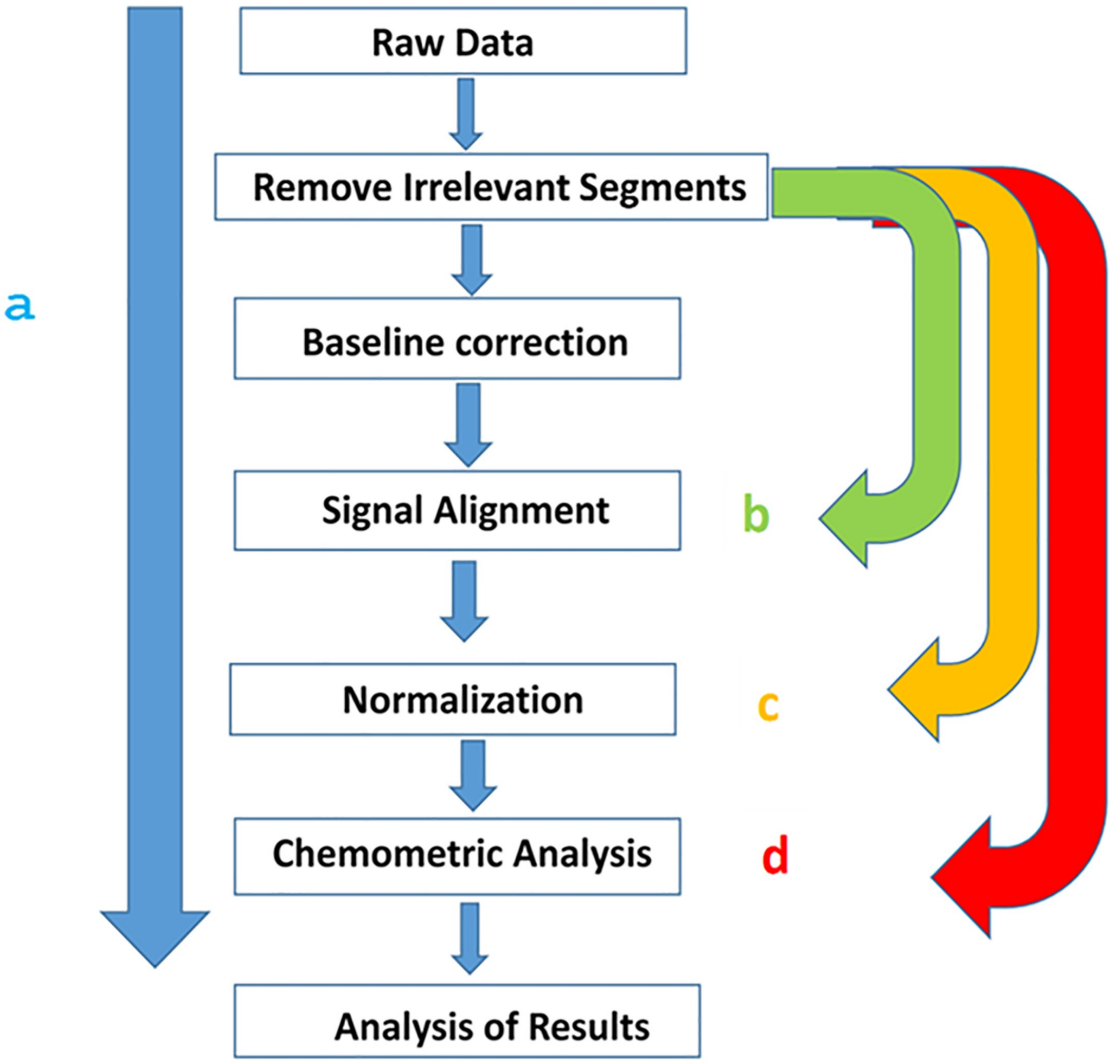
PG-matrix pipeline for the analysis of the PG chromatographic data set. Typically one has to follow the path ‘a’. One can also take path ‘b’ if base line correction is not required. Path ‘c’ should be followed if baseline as well as alignment steps are not required. Path ‘d’ should be followed if pre-processing steps are not required and one has to perform chemometric analysis directly on the raw data set.

The objective of the present work is to provide a pipeline along with suitable MATLAB codes for both classifying the bacterial PG profiles and identifying the subjacent structural biomarkers of the variability. Final characterization of these conserved and distinctive PG traits requires additional analytical methodologies such as MS and/or NMR. This bioinformatic pipeline is baptised as PG-metrics and will further be helpful towards a better understanding of the diversity and functionality of bacteria’s cell wall chemical biology. To illustrate and optimize the methodologies, we have used both computationally simulated and real life data. The proposed pipeline has been summarized in Fig 1.

## Materials and methods

As discussed above, the data analysis work flow is bottlenecked by the issue of the miss-alignment of the chromatographic data sets. In literature, a few alignment approaches are reported, e.g. correlation optimised warping (COW) [25–27], interval correlation shifting (icoshift) [28–30], msalign a MATLAB-bioinformatics tool [31], dynamic time warping (DTW) [26, 32–34] and parametric time warping (PTW) [35–37]. Each one of these methods have different (i) algorithms to operate on, (ii) computational complexities to deal with, (iii) approaches to select the reference peaks, (iv) alignment parameters to optimize and (v) criteria to evaluate the quality of alignments. Thus, the selection of the alignment approach can have a significant impact on the entire *in silico* analysis. In principle, the selected algorithm must ensure the correction of retention time drifts that leads to the synchronisation of the chemical information, should not introduce artefacts in the shape of the chromatographic profiles and preferably, should be computationally economical. The present work studies the various aspects that need to be carefully handled while carrying out the different alignment procedures.

Three of the most commonly used methods that are quite diverse in their alignment approaches are: (i) COW, (ii) icoshift and, (iii) msalign. These methods are first compared on simulated chromatographic profiles displaying complex drifts in peak retention times and the optimal methods is further used for the alignment of the chromatographic profiles acquired for bacterial cell walls.

### Correlation optimised warping (COW) algorithm

**Fig 2:**
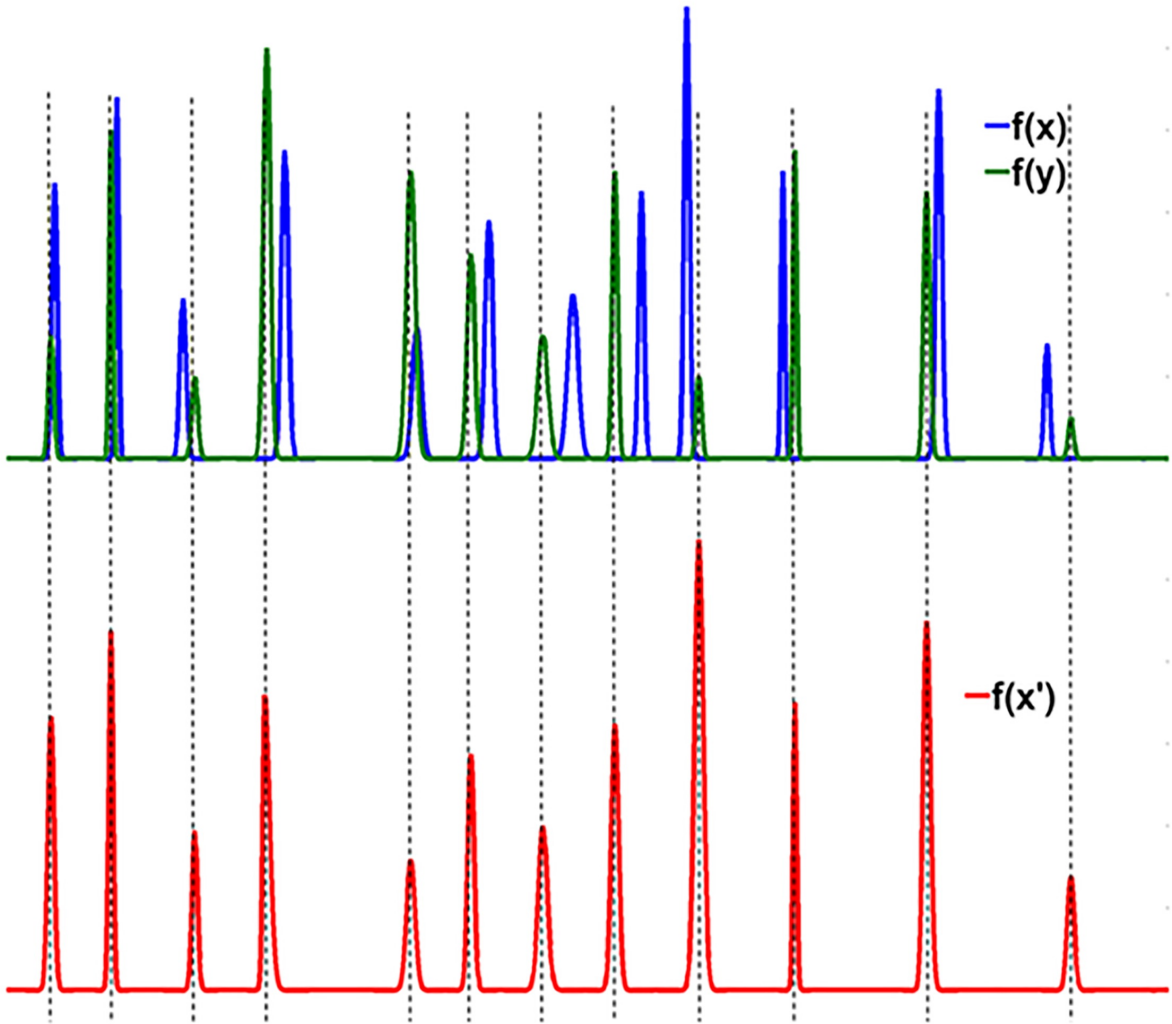
Explaining alignment approach. The unaligned chromatogram f(x), target chromatogram f(y) and aligned chromatogram f(x’).

**Fig 3:**
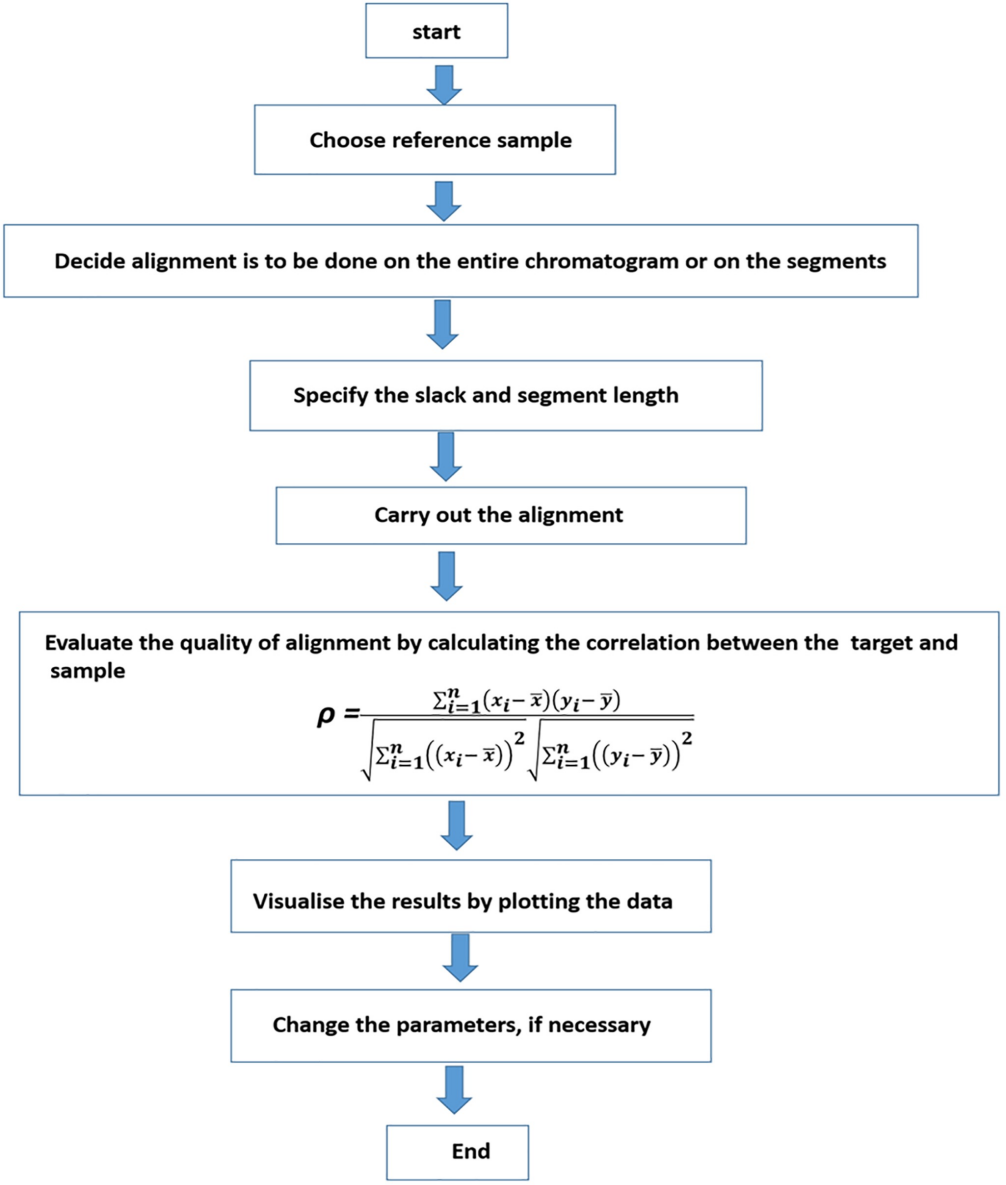
Schematic of COW algorithm. The algorithm requires a reference chromatogram, slack and segment length to carry out the alignment. The quality of alignment is evaluated using correlation between the target and the sample chromatogram.

Correlation optimised warping (COW) has been the most favourite chromatographic peak alignment approach in recent years [25–27]. Based on the expansion and compression model, application of COW ensures peak alignment without introducing any artefacts in the chromatographic profile and thus, has been considered one of the most used trustable alignment techniques. In principle, COW involves two steps: (i) selection of a reference chromatogram and, (ii) segmentation of unaligned chromatograms followed by expansion and compression of each segments to achieve the best alignment. To understand this, Fig 2 shows two simulated chromatographic signals f(x) and f(y) collected over the same retention time period (equal length L). One of these two signals, e.g. f(y), is taken as the reference signal and hence, f(x) is required to be aligned to it. Both signals are divided into N segments of length m (= L/N). Each of the N segments of f(x) can be warped to smaller or longer length with a linear interpolation approach. The amount of warping is controlled by a parameter called slack, denoted as t. In order to make sure that initial and final time points of the chromatograms retain their position, the first segment can only be warped to the right side and similarly, the last segment can only be warped to the left side. All the remaining N-2 segments can be warped in both the directions in the range [-t, +t]. In essence, the alignment of two chromatograms f(y) and f(x) boils down to finding the optimum warping parameters for each of the N segments. Having achieved these parameters for warping f(x), the warped signal f(x´) can be safely approximated to the reference f(y) chromatogram (Fig 2). The quality of the alignment is evaluated by calculating the correlation coefficients (ρ) between the target and warped signal. The scheme of COW is summarised in Fig 3.

### Interval correlation shifting (icoshift) algorithm

**Fig 4:**
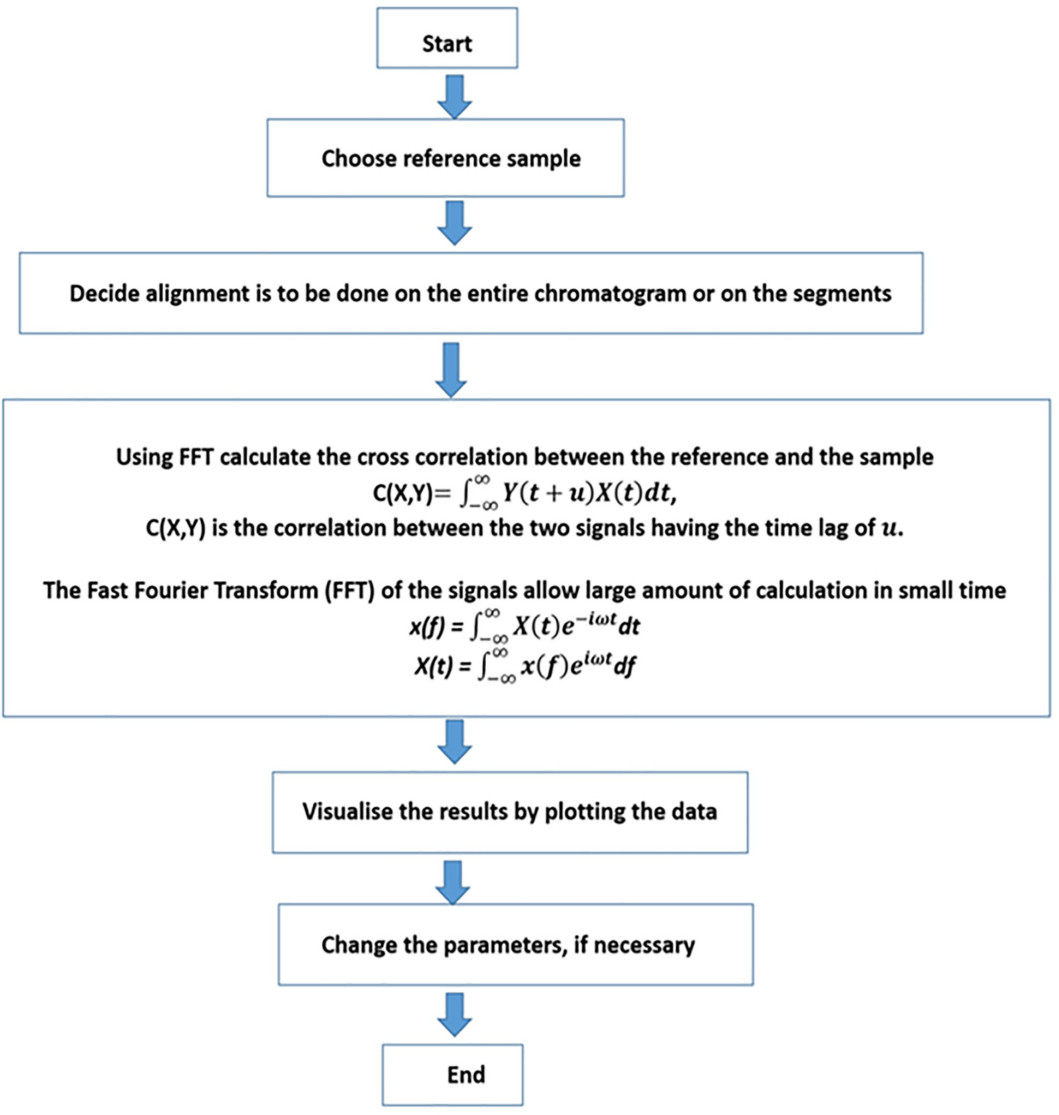
Schematic of icoshift algorithm. The algorithm requires a reference chromatogram, and the range over which the alignment is to be carried out. The quality of alignment is evaluated using cross correlation between the target and the sample chromatogram.

The alignment of two chromatograms with icoshift algorithm [28–30] involves two steps: (i) selection of a reference chromatogram and, (ii) insertion and deletion of certain segments to achieve the best possible alignment with the reference chromatogram. A clear difference between COW and icoshift lies in the second step, the former works with compression and expansion approach whereas latter works on insertion and deletion approach. To understand the working mechanism of the icoshift algorithm, we again consider the pair of unaligned chromatograms f(x) and f(y), shown in Fig 2. In order to align f(x) to the reference chromatogram f(y) a warping parameter μ in the time-shift interval [-t, t] is selected and used to generate a warped function f(x´) = f(x+ μ). A cross correlation term C(μ) is evaluated that measures the extent of integral overlap between f(x´) and f(y) for a selected value of μ. The warping parameter is considered as the optimal if it maximises C(μ). The cross correlation term is evaluated using the fast Fourier transform (FFT) engine. The scheme of icoshift is summarised in Fig 4.

### Msalign: A MATLAB-bioinformatics tool for aligning the chromatograms

**Fig 5:**
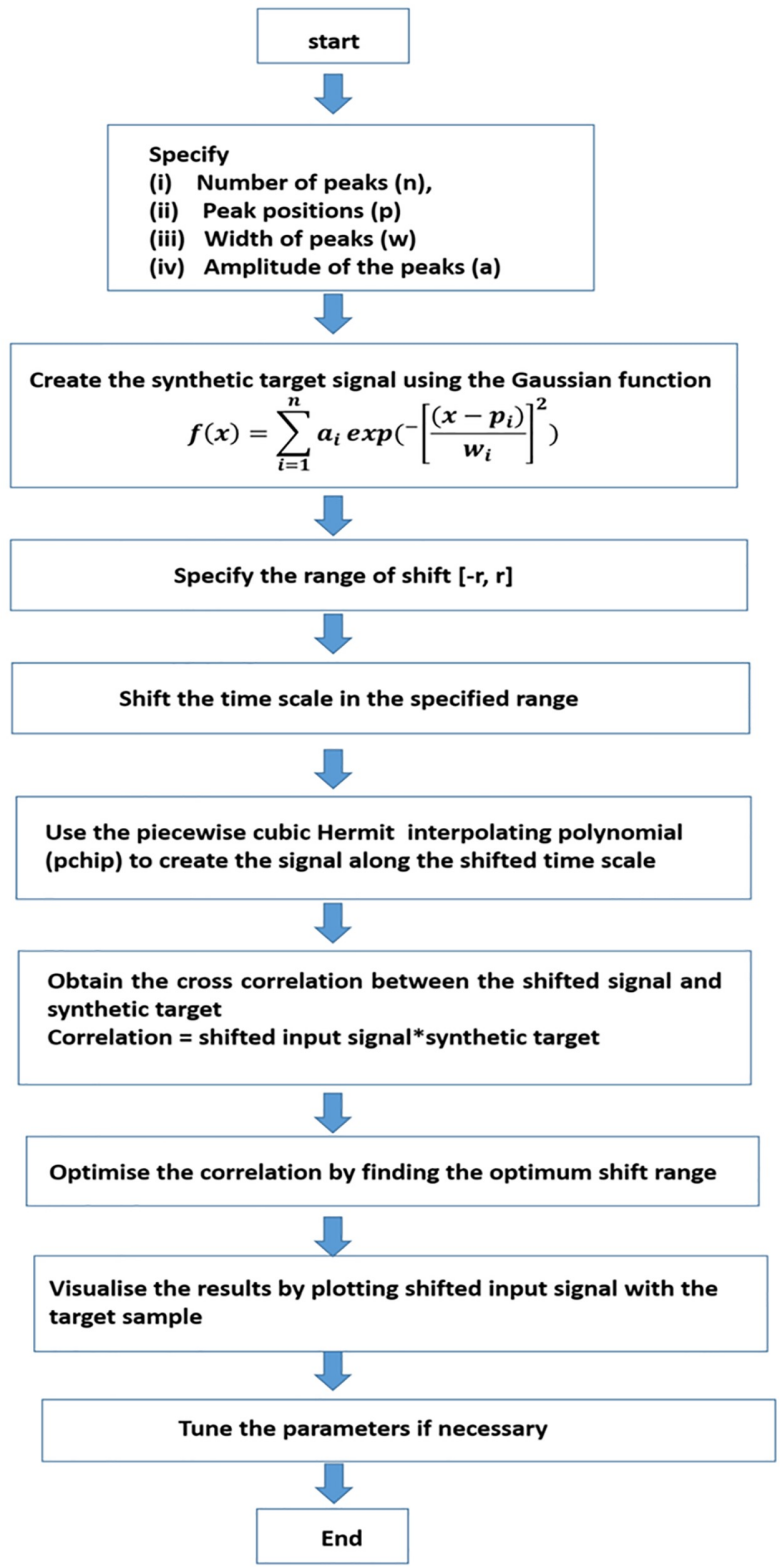
Schematic of msalign algorithm. The algorithm requires a synthetic reference chromatogram, range of shift. The quality of alignment is evaluated using cross correlation between the target and the sample chromatogram.

A chromatographic peak can be approximated using a Gaussian function of appropriate width. Msalign approach takes the advantage of this fact and involves the synthesis a set of reference peaks using Gaussian pulses of suitable widths at desired time points [31]. The time domain of the unaligned chromatogram is shifted to ensure the maximum cross correlation between the chromatographic and the reference peaks. The aligned chromatogram is obtained by performing piecewise cubic interpolation of the shifted chromatogram to the original time points. The cross correlation term is calculated by multiplying the target and the shifted chromatograms over a specified range. The scheme of msalign approach is shown in Fig 5. Compared to COW and icoshift, this approach intrinsically has an advantage that it does not require a reference chromatogram from the sample set.

### Principal component analysis (PCA)

PCA [12–15] decomposes a data matrix X as a product of two matrices, T and P, of smaller sizes. Mathematically, it can be represented with Eq 1

$$X=TP^{T}+E$$ (1)

The dimensions of matrices X, T, P and E are I×J, I×F, J×F and I×J, respectively. Where, I, J, E and F are the number of samples, variables, residual matrix and the number of factors, respectively. The matrices T and P contain the score and loading information. The matrix T explains how the samples are related with each other while matrix P explains how the variables are related with each other. E contains the information that remained unexplained by the PCA model. The superscript T indicates the transpose of the matrix. The matrix factorization of X is carried with certain constrains, for example, the obtained T and P matrices must be orthogonal and orthonormal matrices, respectively. Mathematically, these constrains are represented with given below equations

$$T^{T}T=D$$ (2)

$$PP^{T}=I_{J}$$ (3)

D is the diagonal matrix of dimension I×I with non-zero values along the diagonal and zeros at off diagonal positions. I_I_ is a unitary matrix of dimension J×J that contains value of one at along the diagonal and zero at off-diagonal positions.

The factorization of the matrix X with above explained constrains could be achieved with different approaches. For example (i) NIPALS (nonlinear iterative partial least square) algorithm [14], (ii) singular value decomposition (SVD) [14] approach or, (iii) Eigenvalue-Eigen vector decomposition [14]. Among these, SVD is the most commonly used approach to fit a PCA model. The SVD analysis of X essentially generates a diagonal matrix S that contains positive diagonal elements in the decreasing order and the two unitary matrices of U and V. The Eqs 4–6 represents summarises it.

$$X=USV^{T}$$ (4)

$$UU^{T}=I_{J}$$ (5)

$$V^{T}V=I_{J}$$ (6)

The desired T matrix can be obtained by multiplying U by S. The matrix V can be equated to P.

T=USandP=V (7)

In the context of peptidoglycan analysis, PCA will essentially decompose the matrix X (I×J, where I is the number of samples and J is the number of chromatographic retention time points over which the data are collected) that contains the chromatographic data sets for a set of PG samples belonging to different bacteria. This decomposition of PG data set X with PCA will also generate the two matrices T (score matrix) and P (loading matrix). The matrix T will contain information about the PG samples. For example, Two PG samples can be considered as similar if they have similar score values in T matrix. A difference in score values for any given set of PG samples will indicate the difference in the PG composition in the bacterial cell wall. The analysis of loading matrix T that represents the chromatograms can be helpful in finding the set muropeptides features that can be used to characterize the PG composition of the bacterial cell wall.

### Simulation of the data sets for comparing the alignment approaches

The chromatographic profiles were simulated using the given below Gaussian function

$$f(x)=\sum_{i=1}^{n}a_{i}\;{{exp(}^{-}[\frac{\left(x-p_{i}\right)}{w_{i}}]}^{2})$$ (8)

where *a*_*i*_, *p*_*i*_, *w*_*i*_ and *n* defines the amplitude, peak position, width and number of the simulated peaks. In total, 21 chromatographic profiles are simulated with different parameters that are summarised in S1 Table. The simulated profiles differ in three aspects: (i) the peak position, (ii) number of peaks and, (iii) amplitude of the peaks. In order to make the simulated data set more realistic, a baseline offset to the profiles is also added. These intrinsically complex profiles are simulated with an objective to test the alignment efficiencies of the selected methods.

### Acquisition of bacterial peptidoglycan chromatographic data sets

The real life PG chromatographic data sets were collected using ultra-performance liquid chromatography coupled with UV-detector. Eight bacterial PG samples belonging to the class of Alphaproteobacteria were analysed: *Asticcacaulis biprosthecum* (A. bip), *Acetobacter aceti* (A. ace), *Angulomicrobium tetraedale* (A. tet), *Caulobacter crescentus* (C. cre), *Gluconobacter frateurii* (G. fra), *Gluconobacter oxydans* (G. oxy), *Hirschia baltica* (H. bal) and *Thalassospira lucentensis* (T. luc). The protocol used for the PG isolation and chromatographic data collection can be seen in the literature [5].

### Software used

All the analyses are carried out using PG-matrix. The necessary codes for PG-matrix are written in the.m file format of MATLAB platform. The manual is given as DOCX file in the S1 Material. The zip folder contains the MATLAB codes in.m file format that can be seen in supporting information (S2 Material)

## Results and discussion

### Optimization of the data pre-processing to eliminate the artefacts caused by the instrumental fluctuation

**Fig 6:**
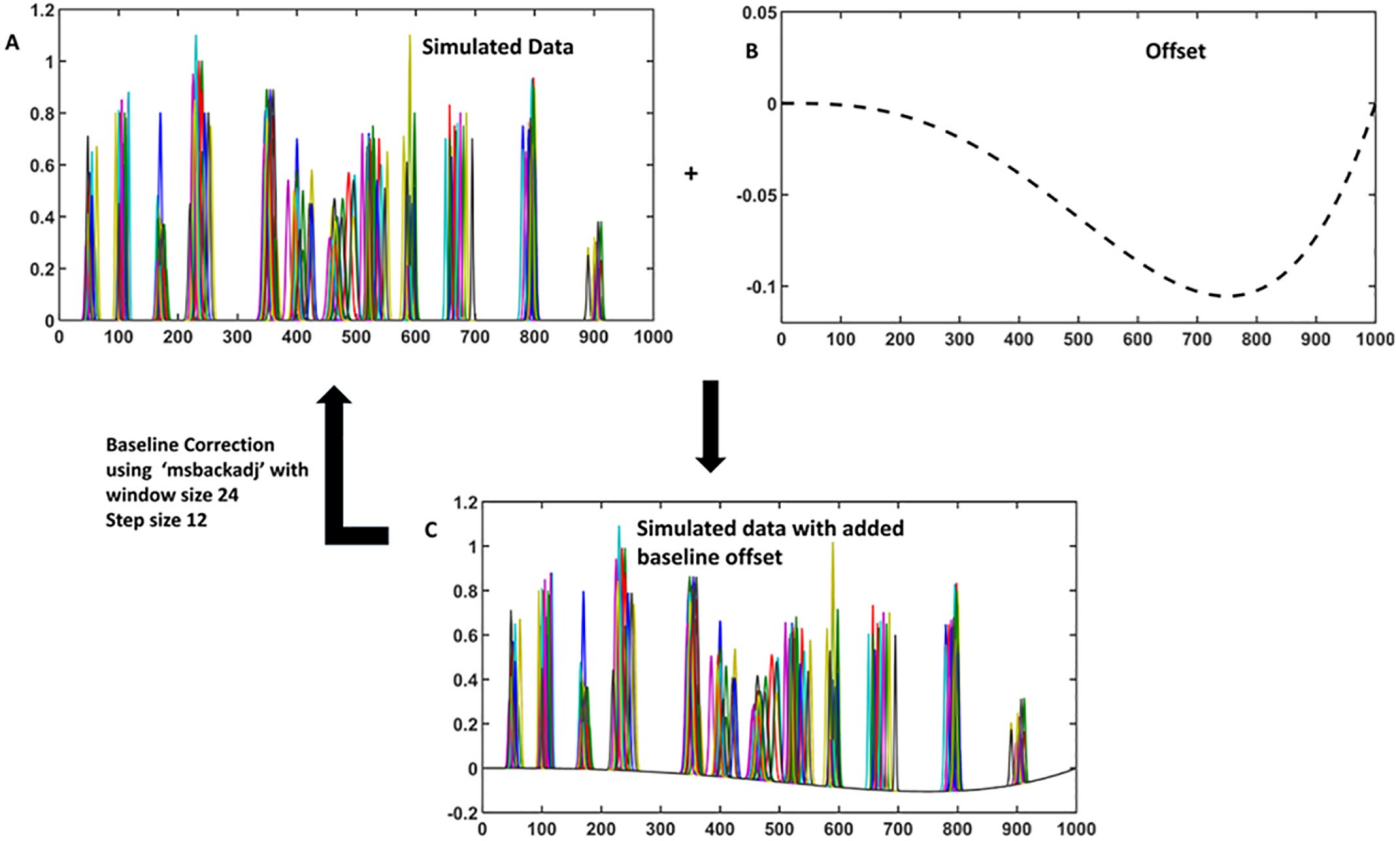
Baseline correction. (A) Simulated profile. (B) Offset profile. (C) Simulated data with baseline offset.

As shown in Fig 1, the first step in the data pre-processing pipeline is the correction of baseline offsets that arises due to the fluctuation of instrumental parameters. An offset from the baseline induces an error to the chromatography intensity values leading to inaccurate quantitative and qualitative analysis of the sample set. There are quite a few methods that can be used to correct the baseline offsets, for example (i) subtraction of a blank chromatogram, (ii) subtraction of a constant value and (iii) subtracting the synthetically created baseline. Over the years, the best method to achieve this has been the subtraction of the synthetically created baseline [38]. It is mainly because the first two methods have limited applicability due to the irregular nature of the baseline offsets from one sample to another. The baseline estimation essentially involves (i) finding the baseline points with an appropriate threshold and (ii) regressing the varying baseline to the estimated baseline points of the chromatogram with a spline approximation over multiple shifted windows of a fixed width. The approach requires optimization of the three parameters: (i) window size, (ii) step size and, (iii) quantile value for estimating the baseline points. The optimization of the parameters is aimed towards achieving the best approximation to ensure offset is taken care without sniping the intensity values. As shown in Fig 6, the simulated profiles are baseline corrected using a window size of 24 and a step size of 10.

After performing the baseline correction, the data needs to be aligned to be comparable on the time axis. As discussed earlier, there are quite a few alignment methods available. In order to optimize the alignment part of the data processing pipeline, first a comparison is carried out among the three selected alignment methods on the simulated data sets. The best method will be further used for the alignment of the real life data set.

### Comparing different alignment approaches with synthetic data

#### Application of the COW algorithm

The baseline corrected data sets are now subjected to the COW alignment algorithm. One of the crucial steps when applying COW is the selection of the reference chromatogram. However, the fact that there is no *a priori* information available makes it difficult to choose an appropriate reference chromatogram. Indeed, application of COW using an inappropriate reference sample can introduce certain artefacts (e.g. peak broadening or peak shrinking) that can bias the data analysis outcome. Therefore, it is important that a proper analysis is carried out before selecting a reference sample. Some of the criteria that can be useful for making the choice are listed below:

1. A chromatogram can be selected as the reference if it is collected under stable instrumental conditions, i.e. the preceding and next chromatograms do not have appreciable drifts in their peak positions.
2. A chromatogram which is drifted appreciably compared to other samples on either side of time axis should not be considered as reference sample
3. A chromatogram selected as the reference must have maximum number of common peaks. In other words, it should be able to explain the actual chemical phenomena taking place.

The selection of a reference known PG sample, or standard, can be a good choice when all the other samples are closely related to it. Often, such information is not available and peak by peak characterization will be required to establish the relationship. The present work address this issue by suggesting the use of a chromatogram having a maximum number of common peaks as the reference sample. Mathematically, it can be evaluated using Eq 9 that involves the evaluation of the correlation coefficient values between the reference and other samples.

$$\rho =\frac{1}{n}(\sum_{i=1}^{I}\frac{{\left(X-mean(X)\right)}^{T}\;(Y_{i}-mean(Y_{i}))}{std(X)std(Y)})$$ (9)

where x is the reference sample, Y_i_ is the i^th^ chromatogram, mean (X) and mean (Y), std (X) and std(Yi) are their corresponding means and standard deviations. Finally, n is the number of samples that need to be aligned.

In the present case of aligning the simulated chromatograms (shown in Fig 6(C)) using COW algorithm, one in principle can select any of the chromatograms from 2–12 and 17–21 as they contain maximum number of peaks. A choice can be easily made based on the cumulative correlation coefficient values between the selected reference chromatogram and other samples. The sample that provides maximum correlation can be selected as the reference chromatogram. The correlation coefficient for different possible reference chromatograms are calculated using the Eq 9 and reported in Table 1. Based on this, the second chromatogram is selected as the reference sample.

**Table 1:** Selecting the reference chromatogram. The average correlation coefficient between the selected reference chromatogram and rest of the samples.

Selected reference chromatogram	Average correlation coefficient (ρ)
S1	0.87
S2	0.97
S3	0.94
S4	0.91
S5	0.92
S6	0.88
S7	0.87
S8	0.92
S9	0.92
S10	0.95
S11	0.89
S12	0.87
S17	0.86
S18	0.91
S19	0.92
S20	0.93
S21	0.91

**Fig 7:**
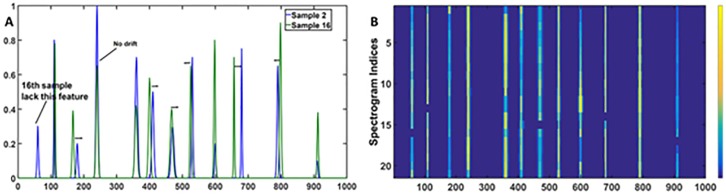
COW alignment. (A)The reference (sample 2) and the unaligned sample (sample 16) indicating the complexity of the miss-alignment. (B) The COW aligned heatmap of the simulated data sets. The results clearly indicate that COW provides an optimum alignment.

To begin with, the sixteenth sample is aligned using different combination of segment lengths and slack parameters. The overlay plot between the sample and the target is shown in Fig 7(A). It can be seen that selected chromatograms have different number of peaks with irregular drifts in their positions. In order to study the effect of segment length variations, the slack parameter is kept constant and the alignment is carried out with different segment lengths. For example, in order to align the 16^th^ chromatogram to the 2^nd^ chromatogram, the slack is fixed at 20 and segment lengths varied from 100 to 30. The quality of the alignments are compared visually as well as using the correlation coefficient between the reference and the aligned chromatograms. The correlation coefficients for different segment lengths are summarised in S2 Table. It can be seen that a good alignment is difficult to achieve using longer segments. As one reduces the segment length it leads to an improvement in the alignment and thus, all the segment lengths in the range 30–40 appeared to work well in aligning the selected chromatogram. Though, the best alignment with maximum correlation is achieved with the segment length of 35. To understand the effect of different slack parameters, the segment length is kept constant and slack parameters are varied. For example, for the optimised segment length of 30, the slack parameters are varied in the range of 1–25. It is found that the quality of the alignment improves with the flexibility provided by the slack parameter. The correlation coefficient for the different slack parameters are summarised in the S3 Table. From these results one can conclude that the maximum cumulative correlation product is achieved with the segment length of 35 and slack of 25. With these optimised parameters, the remaining samples also showed a perfect alignment (Fig 7(B)).

#### Application of the icoshift algorithm

**Fig 8:**
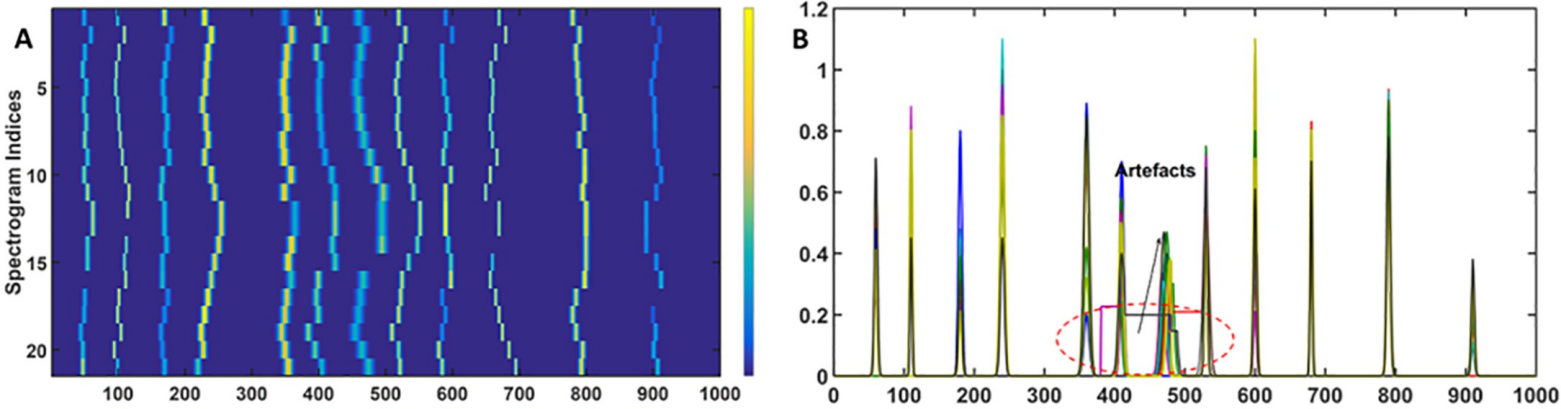
Icoshift alignment. (A) Heatmap obtained from the icoshift analysis on the complete range of simulated data sets and the obtained results are clearly unsatisfactory. (B) The alignment results obtained from the icoshift analysis on segments. The obtained results improve but contain artefacts such as horizontal segments in the middle of the segments and it is difficult to remove because of the complexity in the drifts.

The baseline corrected data sets are subjected to the icoshift algorithm by selecting the second simulated chromatogram as the reference. The icoshift analysis is performed on the entire range of the simulated chromatograms. The obtained results are shown as the heat-map, in Fig 8(A). Before proceeding further, it is important to mention that representation of data using heatmap tool allows simultaneous visualisation of all the peaks of a given sample set in a single plot where the increase in intensity can be observed as the colour in the map changes from blue to yellow. The spectrogram indices in the heatmap indicate the samples. For example, there are 21 simulated chromatograms therefore the index varies from 1 to 21 in the corresponding heatmap, shown in Fig 7B or Fig 8A. The heatmap also allows the visualization of the chromatographic peak alignment in a single plot. If all the chromatograms are aligned (as shown in Fig 7B), the heatmap will essentially consist of vertical lines. If there is misalignment, the heatmap will consist of disjointed lines as shown in Fig 8A.

Thus, it can be seen from Fig 8A that unlike COW (shown in Fig 7B), it is difficult to achieve the alignment of the simulated chromatograms by applying the icoshift algorithm on the entire chromatographic range. In order to overcome this problem, icoshift is applied piecewise on the different segments of the chromatograms. The entire chromatographic range is split into eleven segments [1–80, 81–141, 142–201, 202–280, 281–380, 381–480, 481–560,561–610, 611–710, 711–810, 811–1000], and each of them are separately aligned. The aligned pieces are joined together, and shown in Fig 8(B). The results appeared to improve significantly as one goes from the complete to piecewise icoshift analysis. All the segments except for the 6^th^ segment (381–480) are found to be aligned to the reference sample. Based on our results, even though icoshift is computationally economical, COW seems to work much better.

#### Application of the msalign algorithm

The msalign algorithm was initially introduced for aligning mass spectra. However, by optimising the warping parameters it is possible to align the chromatographic peaks. msalign essentially involves synthesis of reference sample by simulating the Gaussian peaks at the desired positions. In the present work, the Gaussian peaks are simulated at the median peak position across the sample set to optimise the alignment efficiency of the msalign algorithm. However, not to bias the analysis, one has to use expert knowledge in PG chromatography to synthesise the reference chromatograms. It is important to mention that similar to icoshift, this approach is also computationally economical. It is due to the fact that msalign also uses cross-correlation approach for performing the calculation that makes it computationally economical [28–31].

**Fig 9:**
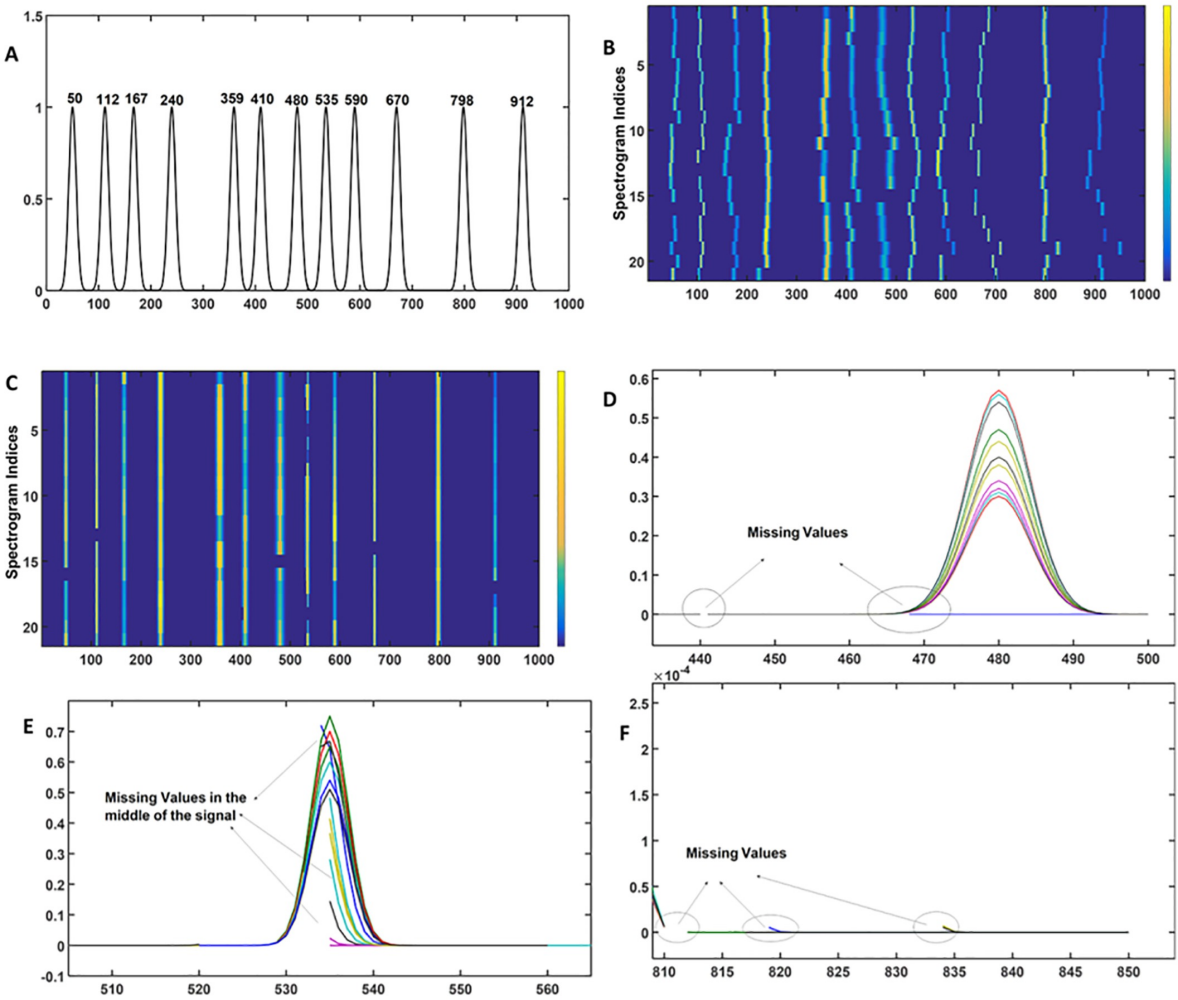
Msalign alignment. (A) The synthetic target with pulses centred at desired locations, the synthetic target is generated using the Gaussian function. (B) Heatmap of the simulated data sets using the msalign algorithm, as with the previous case the obtained results are clearly unsatisfactory. (C) Heatmap of the data sets obtained from msalign analysis on different segments. (D), (E) and (F) clearly show that msalign generates artefacts i.e. number of segments even in the middle of chromatogram with no data (i.e. missing values).

The msalign tool requires the user to mainly specify: (i) peak position for the reference peaks and (ii) range of shift. A synthetic target containing the reference peaks at the following position 50, 112, 167, 240, 359, 410, 480, 535, 590, 670, 798 and 912, is generated and shown in Fig 9(A). The msalign algorithm is executed with a range of shift [–50, 50]. The obtained results shown in Fig 9(B) indicate that as for icoshift, a piecewise alignment would be a better strategy to align the signals. The chromatograms are divided in twelve segments [1–80, 81–141, 142–201, 202–280, 281–380, 381–440, 441–520, 521–560, 561–640, 641–710, 711–810 and 811–000]. The obtained results are shown in Fig 9(C). It can be seen that for a number of peaks, a reasonably good alignment is achieved but for instance, in the range of 550–650 the obtained results are far from the optimum. Moreover, there is another problem of missing values that appear right in the middle of the peak. In order to show it clearly, problematic segments are zoomed and shown in Fig 9D–9F. The problem of achieving an accurate alignment and the generation of missing values limits the applicability of msalign approach.

Before proceeding further it is important to mention that application of icoshift and msalign algorithms on the entire chromatogram failed to provide the desired alignment. It is mainly due to the complexity of the drifts in the peak positions. Both algorithms, instead of providing peak by peak alignment, look for overall alignment (i.e. works towards achieving the global solution). Therefore, an approach that involves alignment of the individual peaks can optimise the efficiency of these two algorithms. To achieve this, chromatograms were divided into several segments of unequal length and these algorithms were applied on each segment separately. Finally, each of the aligned segments were joined together to create the entire aligned chromatogram. For icoshift the optimum alignment was achieved by dividing the entire chromatograms into 11 segments and for msalign the optimum alignment was achieved by dividing the entire chromatograms into 12 segments. The optimum number of segments were achieved by performing the analyses on different number of segments and evaluating the quality by calculating the cross-correlation coefficient.

Comparison of these three most commonly used methods suggests that compared to COW, icoshift and msalign are computationally more economical. Plus, msalign does not require any reference chromatogram that provides a clear advantage over other two methods. Icoshift generates artefacts that may bias the outcome of the workflow. Msalign can generate missing values right in the middle of the peak thereby distorting the shape of the chromatographic profiles. Taken together, one can infer that COW algorithm provides the more reliable method to align the peaks. Thus, in the reported PG-metrics pipeline, we select COW algorithm for real life samples.

### PG-metrics work flow for the analysis of Alphaproteobacteria data set

**Fig 10:**
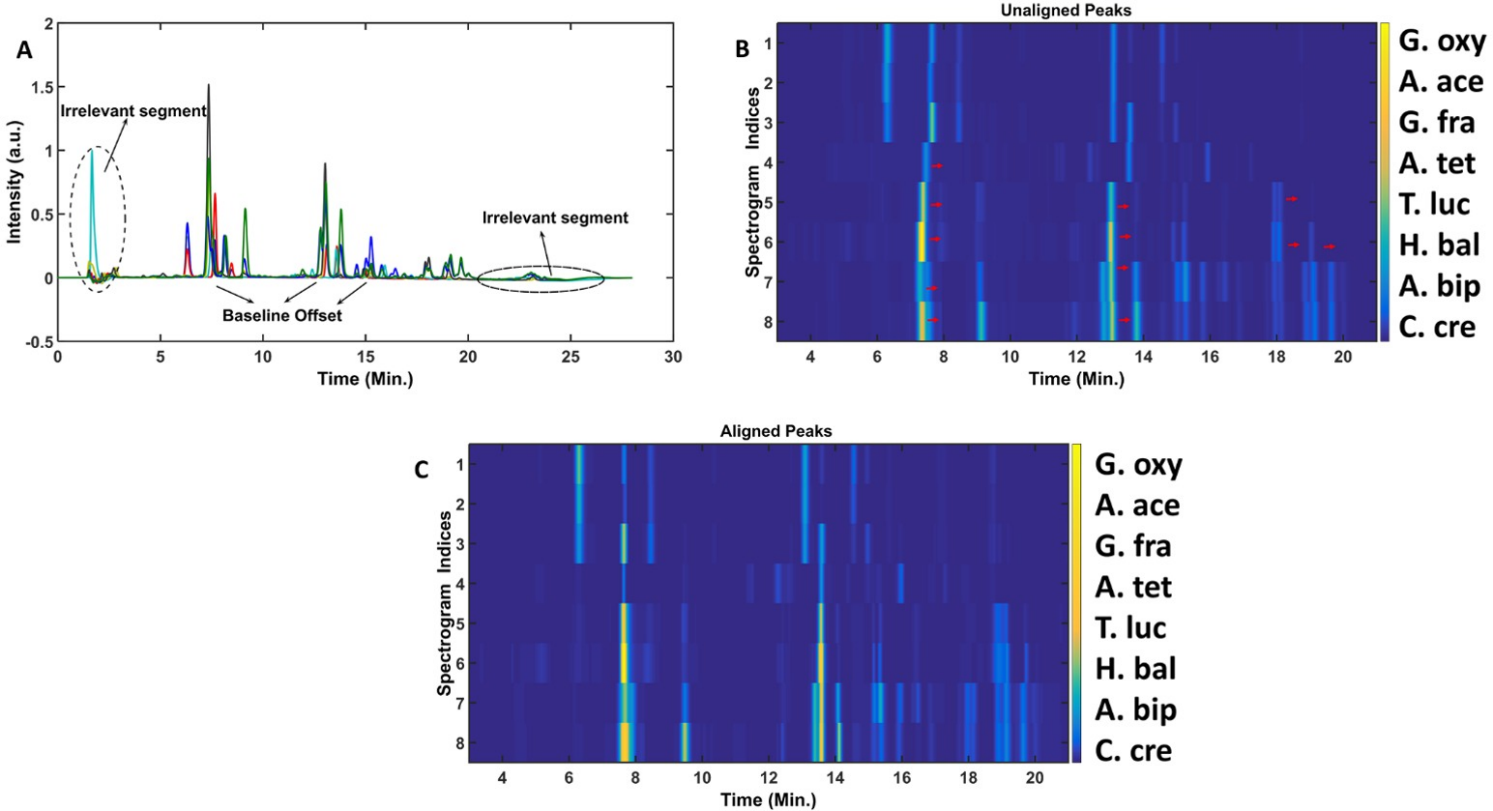
Pre-processing the raw data. (A) The raw chromatograms of PG samples belonging to the class of Alphaproteobacteria, the chromatograms contain (i) the irrelevant segments in the beginning and towards end of the gradient and (ii) the baseline offset. (B) The Heatmap of the PG samples after removing the irrelevant segments in the region (<3 min. and >21 min.) and correcting the baseline offset, the arrow (→) indicate the direction of shift required to achieve the alignment fitting to biological premises. (C) Heatmap of the COW aligned chromatograms.

The chromatograms of the Alphaproteobacteria samples are shown in the Fig 10(A). A detailed description of the chemical composition of these PG samples has been recently reported [5].

Details on the sample and data acquisition (growth conditions, UPLC utilized, chromatographic conditions, etc.) could be seen in the work carried out by Espaillat et al. [5]. The chromatograms, apart from having baseline offset and retention time drifts, also contain certain irrelevant segments that do not contain any bacterial cell wall related information. The irrelevant segments are (i) solvent fronts appearing over the time range of 1–3 min and (ii) column wash appearing over the time range of 21–26 min. The irrelevant segments can be safely trimmed out to prevent any undesirable effect on the subsequent chemometric data analysis workflow. The trimmed data is subjected to baseline correction with quantile value of 0.1, window size of 100 and step size of 100. The data is next subjected to COW analysis. The complexity of the retention time drifts, as shown in Fig 10(B), makes it difficult to perform the alignment on the entire chromatographic profiles. Therefore, the entire chromatogram is divided into different segments [1–450, 451–800, 801–1082]. Each segment is then separately aligned to the selected reference chromatogram with optimum segment length and slack parameter. For example, sample 3 is chosen as the reference sample and sample 1 is aligned to it with the following scheme: Alignment of first segment [1–450]: segment length = 45 and slack = 20; Alignment of second segment [451–800]: segment length = 32 and slack = 12; Alignment of third segment [801–1082]: segment length = 22 and slack = 7. Similarly, all the chromatograms are aligned using proper combinations of the segment length and slack parameter. The aligned chromatograms are shown in the Fig 10(C). Even though there are algorithms to correct the retention time drifts of the chromatograms, it still needs the expertise and intuitiveness of the user. The baseline and drift-corrected data sets are further normalized to unit area to ensure the technical variabilities are taken care.

Sample pre-processing (i.e. baseline correction and peak alignment) is not just important for the subsequent chemometric analysis of the entire chromatogram but also ensures minimization of the errors associated to specific muropeptides (i.e. comparing the cross-linkages or average glycan strand lengths in different bacteria). The proper pre-processing can automatize the cell wall characterization.

### Principal component analysis (PCA): Finding groups, biomarkers and interesting candidates

**Fig 11:**
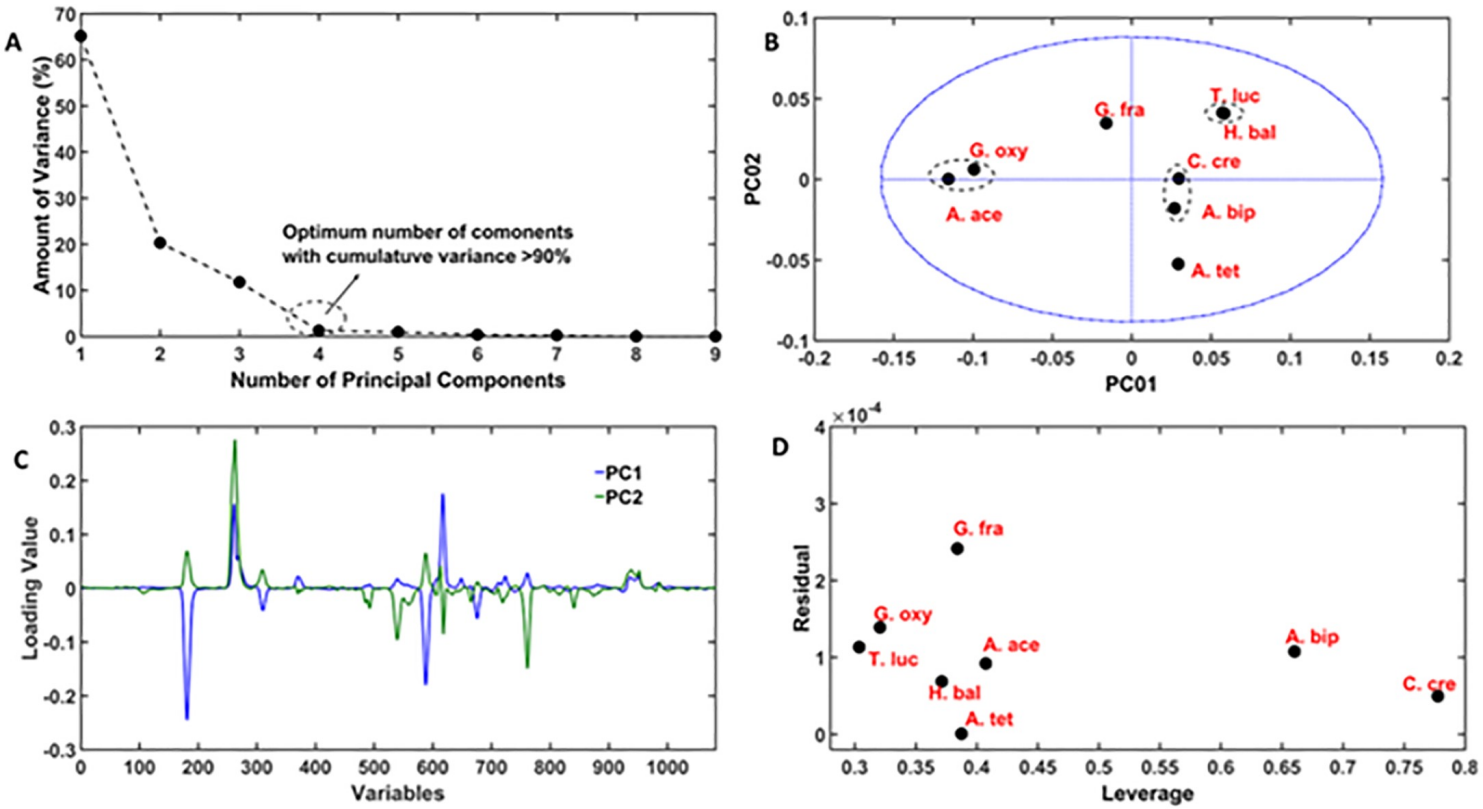
PCA on pre-processed data sets. (A) Amount of variance versus number of principal component plot indicating 4 as the optimum number of factors for PCA. (B) PC1 and PC2 score plot classifying the samples. (C) PC1 and PC2 loading vector plot indicating the features in the chromatograms responsible for the obtained classification. (D) Leverage versus residual plot for finding the outlier samples. The plot predicted G. fra, A. bip and C. cre as the interesting candidates in the analysed bacterial sample set.

The pre-processed data set is subjected to PCA for finding the natural grouping of the samples and also interesting candidates in the sample set based on their atypical PG profiles. Before fitting a PCA model, the first thing that has to be carried out is finding the optimum number of principal components (PCs) not to over- or under- fit the data sets. The best way of finding the rank is plotting the amount of variance captured by each principal component against the principal component number. As it can be seen in Fig 11(A), one can conclude that a 4 PCs model (90% variance) is optimum to capture all the important information buried in the data set. A score plot that explains the variation of the score values along the different PCs are plotted. For a 4 component PCA model there are six PCA score plots that can be created. For example, PC1 versus PC2, PC1 versus PC3, PC1 versus PC4, PC2 versus PC3, PC2 versus PC4, and PC3 versus PC4. In principle, each of them should be carefully studied for finding the relationship among the samples. Though, it is true that not all of them are equally important in describing the chemical phenomena taking place in the samples. In the present case, among these 6 combinations PC1 versus PC2 score plot, shown in Fig 11(B), provides the correct classification of the samples. From the score plot, it can be seen that (i) G. oxy and A. ace, (ii) T. luc and H. bal and (iii) C. cre and A. bip form three separate groups in three different quadrants of the score plots whereas, A. tet and G. fra do not belong to any group.

The PCA also provides the loading vectors that explain how the variables are related with each other. The study of loading vector plots can help in finding the set of the variables that can explain why the samples belong to a particular group and what make them interesting within a sample set. The loading vector plots corresponding to the PC1 and PC2 are shown in Fig 11(C). The samples with higher PC1 and PC2 score values irrespective of sign (positive or negative) weigh heavily to PC1 and PC2 loading vectors, respectively. The loading vector of PC1 gives a clear idea about the negative correlation among certain features in the chromatographic profiles. All the features with negative loading values can mainly be seen in the samples having the negative score values along the PC1 axis. The samples having positive PC1 score value either lack these features or contain them only in very small abundance. There are certain features with positive loading values (in the range 600–900 corresponding to 13–20 min) in PC1 loading vectors can that only be seen in the samples having positive score values. Thus, PC1 loading vector clearly explain the classification of the samples in two groups (i) A. ace, G. oxy and G. fra and (ii) T. luc, H. bal, C. cre, A. bip and A. tet. The loading vector corresponding to PC2 score mainly reflects the variations of the minor peaks, particularly in the variable range of 600–900. The features corresponding to positive loading values in PC2 loading profile can predominantly be seen in the chromatograms of the samples having positive PC2 score values. Similarly, peaks that correspond to the negative values of PC2 loading vector can be found in the samples with negative PC2 scores values. The PC2 loading vector clearly explain within group classification of the samples.

Finding outliers in the sample set is another task of chemometric based data analysis. An outlier sample can be defined as the one that has obvious differences from others which, in the case of PG samples, means either cell wall with distinctive muropeptides or with significant variation in the relative abundance between common muropeptides. Such samples in PCA model can be identified as the ones that are either not well described by the models or they influence the model too much. Such samples can be found using two statistical parameters: residual and leverage [13]. A sample that is not well described by the model will have unusually high residual and the samples that have high influence on the model will have unusually high leverage. The residual of the samples can be calculated using the given below equation.

$$Q=EE^{T}$$ (10)

The diagonal elements of Q matrix contains the residual of the samples. For example, first diagonal element explain the residual of the first sample and so on. The leverage can be calculated using the given below equation.

$$L=T{\left(T^{T}T\right)}^{-1}T^{T}$$ (11)

The diagonal elements of the matrix L describe the leverage of the samples. The first diagonal element explain the residual of the first sample and so on. All the samples having either leverage values or residual values or both greater than 0.75 quantile limit can be considered as an outlier. The residual and leverage plot for the analysed Alphaproteobacteria samples are shown in Fig 11(D). Among the selected samples, G. fra has a residual value greater than 0.75 quantile limit. A. bip and C. cre have leverage values greater than 0.75 quantile limit. These three samples are classified as outliers.

The high residual value of G. fra unusually can be attributed to the presence of chromatographic signal appearing at time point of 8.5 minutes, shown in Fig 10(C), whereas the high leverage values of A. bip and C. cre can be associated to the highly intense peaks in their chromatograms shown in Fig 10(C). Collectively, the developed PCA model classify the bacterial samples based on their relative similarities of the PG compositions. The model also allow finding the group of muropeptides that are responsible for the obtained classification.

### Agreement analysis

**Fig 12:**
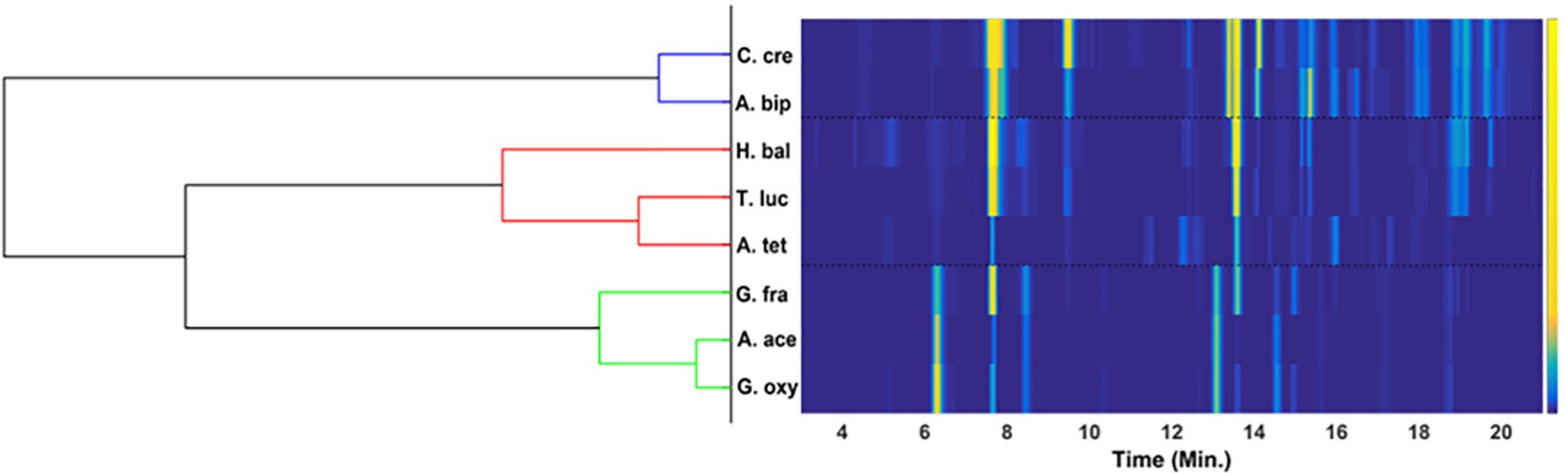
Agreement analysis. Dendrogram obtained from the hierarchical cluster analysis using the Ward linkage criteria and Euclidean distance metric approach. Heatmap of the samples, arranged according to the nodes of the cluster. The obtained results are in agreement with those obtained from PCA model.

It is important to mention here that different chemometric methods have different algorithms, different assumptions for the data sets and, different functions to optimize. To ensure the obtained results are not biased by the choice of chemometric method used, it is convenient to run an agreement analysis by performing a different type of chemometric analysis [39]. For example, one can use (i) neural network analysis [13, 40], (ii) factor analysis [41] (iii) cluster analysis [42] or (iv) probabilistic latent semantic analysis [43] etc. Among these methods, the cluster analysis is the most commonly used approach for finding natural grouping of the samples. In the present work, data sets of Alphaproteobacteria samples are subjected to the Ward clustering with Euclidean distance matric approach for finding the agreement with the results obtained from PCA. The ward clustering results with the corresponding heatmap are shown in Fig 12. The dendrogram mainly consist of three clusters: (i) C. cre and A. bip, (ii) T. luc, A. Tet and H. bal, and (iii) G. oxy, A. ace and G. fra. The obtained dendrogram also indicates that G. fra and H. bal have differences within their corresponding clusters. The obtained clustering results are in true agreement with PCA results. It can clearly be seen and verified that obtained PCA results are genuine and not a consequence of the algorithm used for analysing the data sets.

### Application of PG-Metrics on the peptidoglycan data sets of bacterial mutants

**Fig 13:**
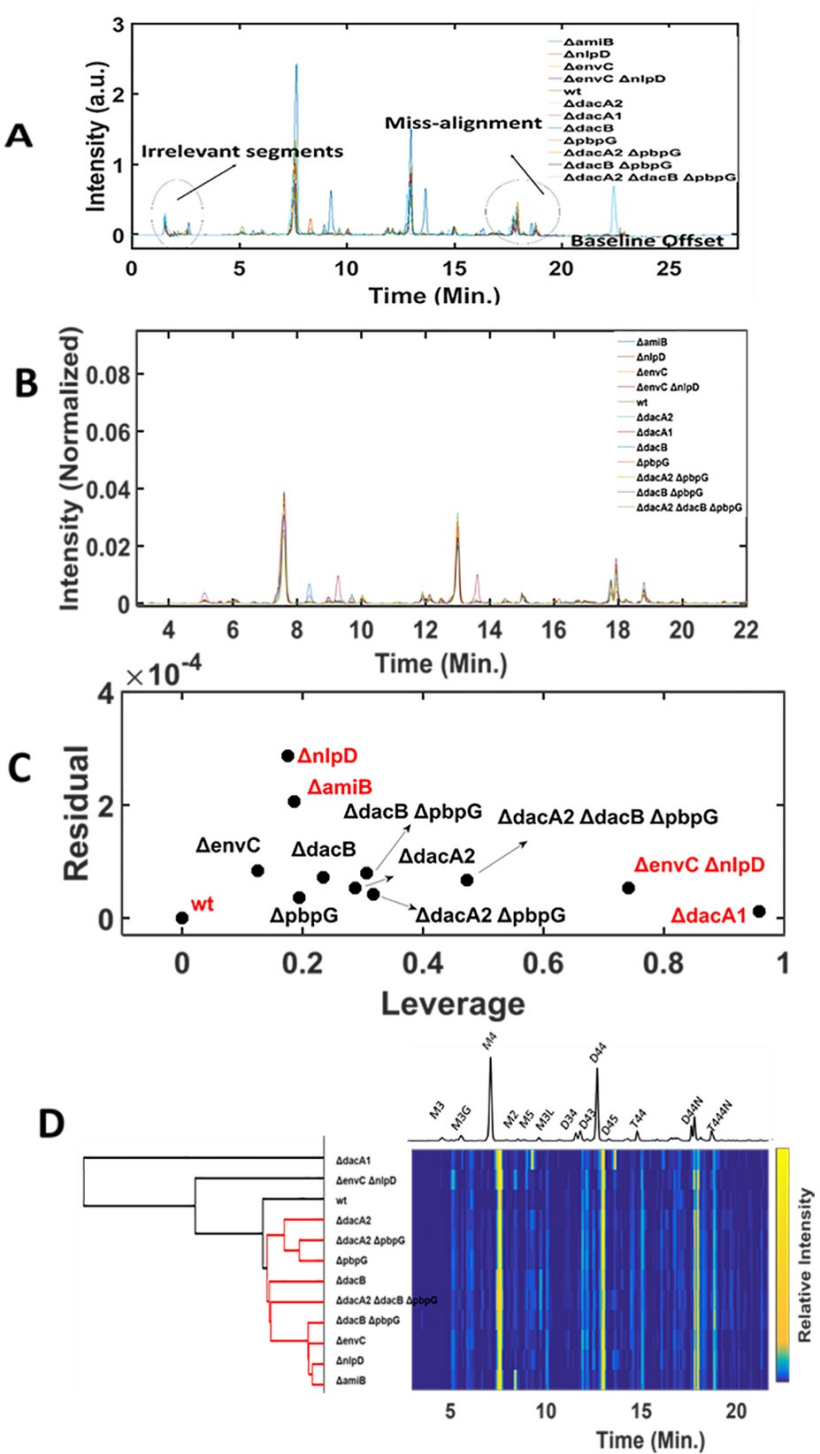
Application of PG-metrics on *Vibrio cholerae* mutants. [A] The raw chromatograms of the *Vibrio cholerae* mutants, [B] pre-processed chromatograms (i.e. removal of irrelevant segments, baseline corrected, COW aligned and normalised), [C] Leverage versus residual diagnostic plot for finding the interesting candidates. [D] Dendrogram obtained from the hierarchical cluster analysis using the Ward linkage criteria and Euclidean distance metric approach. Heatmap of the samples, arranged according to the nodes of the cluster.

**Fig 14:**
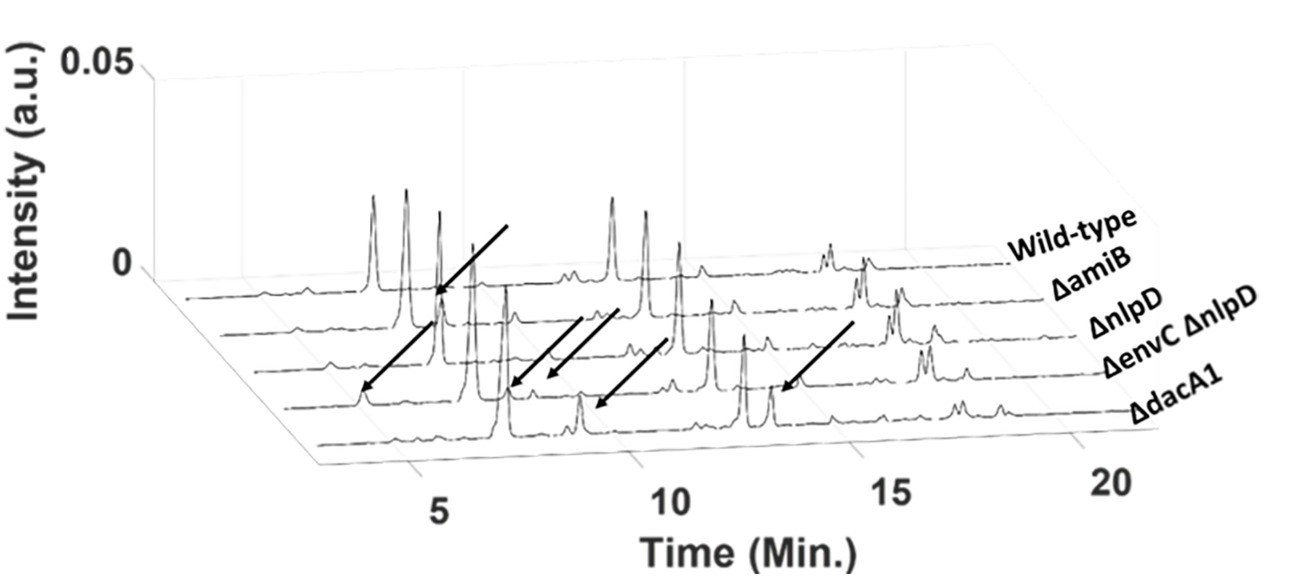
Comparison of the wild-type and PG-metrics predicted outliers. The plot clearly shows the features (indicated with →) in the PG composition of *ΔamiB*, *ΔnlpD*, *ΔenvCΔnlpD*, and *ΔdacA1* that makes them different from wild-type.

In order to further demonstrate the utility of the PG-Metrics, we applied this pipeline for classifying and identifying the subjacent structural variability in a PG chromatographic data sets composed of 12 *Vibrio cholerae* mutants that have been previously characterized [44, 45]: *ΔamiB*, *ΔnlpD*, *ΔenvC*, *ΔenvCΔnlpD*, wild-type (C6706), *ΔdacA2*, *ΔdacA1*, *ΔdacB*, *ΔpbpG*, *ΔdacA2ΔpbpG*, *ΔdacBΔpbpG*, *ΔdacA2 ΔdacBΔpbpG*. The Details on the sample and data acquisition (growth conditions, UPLC utilized, chromatographic conditions, etc.) could be seen in the work carried out by Möll A et al. [44, 45].The application of PG-metrics towards classification of mutants with subtle PG variation would clearly test the potential of the proposed approach. In other words, it will evaluate if PG-metrics can capture even minor variations to subsequently classify the samples. The raw chromatograms of these samples are shown in Fig 13(A). As discussed earlier, removal of irrelevant segments and baseline correction are inevitable steps and are carried out using the suitable codes available in the PG-metrics toolbox. The alignment of baseline corrected chromatograms are carried out using the COW approach by selecting *ΔdacA2ΔpbpG* as the reference chromatogram that has all the common peaks of the analysed samples. It is to be noted that there are other samples that contains the several common peaks and in principle they could also be considered as the reference chromatogram. The optimum segment length (‘m’) and slack (‘t’) parameters for achieving the alignment of rest of the chromatograms with reference sample is found to be 50 and 10, respectively. The aligned chromatograms are shown in Fig 13(B). The aligned chromatograms were further normalised to unit area. As discussed earlier, these pre-processing steps ensures that errors introduced during sample preparation and instrument fluctuation are taken care. The wild-type chromatographic data was subtracted from the data of all other mutants. This step will ensures an unbiased comparison of the selected mutants from the wild-type. The wild-type subtracted data set is further processed for chemometrics analysis. The optimum number of principal components for PCA was found to be four, obtained by plotting the amount of variance against the principal component number. The 4 components PCA model accounts for more than 85% variance indicating all the important variation present in the data set has been explained. The outlier diagnostic plot that describes the variation of residual and leverage values for each sample, shown in Fig 13(C), clearly indicate that four of the samples (*ΔamiB*, *ΔnlpD*, *ΔenvCΔnlpD*, and *ΔdacA1*) were interesting candidates. The two samples *ΔamiB* and *ΔnlpD* presented residual values greater than 0.75 quantile limit whereas *ΔenvCΔnlpD*, and *ΔdacA1* have leverage values greater than 0.75 quantile limit. The obtained results were in agreement with the literature describing that these mutants have distinctive PG composition compared to wild-type [44, 45]. The pre-processed data sets are further subjected to agglomerative cluster analysis with Ward linkage and Euclidean distance matric approach for partitioning the analysed samples based on their relative dissimilarity. The obtained dendrogram along with heatmap is shown in Fig 13(D) and it can be seen that obtained clustering correlates well with results obtained from PCA results and those reported in literature. The individual chromatograms and their comparison with wild-type is shown in Fig 14. It can be seen that *ΔdacA1* mutant has unusual PG composition with relatively high abundance of M5 (monomer pentapeptide) and D45 (dimer tetrapeptide-pentapeptide) that makes it as one of the prime outlier in leverage versus residual plot. *ΔamiB*, *ΔenvCΔnlpD* were known to have similar muropeptide composition but contained M2, M3 and M5 monomers in distinct proportions which made them different from the wild type. The mutant *ΔnlpD* had significantly high amount of overall PG content compared to wild type. The obtained results clearly show that PG-metrics can capture even subtle variations present in the certain cell wall associated bacterial mutants.

## Conclusions

Here, we report PG-metrics, a novel pipeline for performing chemometric analyses of chromatographic PG data sets. Comparison among the three intrinsically different alignment approaches pinpointed COW as the most appropriate method to align chromatograms that have complex drifts in the peak position. The pipeline is successfully validated by analysing the PG of diverse bacteria species belonging to the class Alphaproteobacteria and also of different *V*. *cholerae* cell wall related mutants. The analysis clearly provides all the required information for finding the relationship between the cell walls of the analysed samples.

Even though UPLC provides an outstanding resolution to separate PG subunits, certain muropeptides might still co-elute. Therefore, although PG-metrics represents a simple method for classifying PG data sets, pinpointing potential conserved and distinctive muropeptides amongst samples, MS is still required to prove their chemical structure.

Altogether, chemometrics-based methods are appropriated tools to classify PG from different bacteria based on their potential chemical similarities and differences. This type of classification could be contrasted with the existing bacterial phylogenetic organization to investigate the evolutionary emergence and prevalence of certain PG modifications. Comparative analysis of taxonomic and PG markers can provide insights about the conserved PG determinants along certain bacterial phylogeny branches and those which, by contrast, have been externally imprinted by specific environmental pressures within a particular group. In this regard, PG quantitative studies of bacteria cultivated under a diverse conditions can also provide valuable information about the PG chemical and structural plastic response to changing environments.

Finally, the analysis of the PG of mutants in cell wall-associated activities can be instrumental to find the functionality of these activities in PG biosynthesis and remodelling and would be particularly useful for the analysis of mutant libraries. Peptidoglycan analyses of such mutant collections can reveal the genetic determinants and regulatory pathways of PG homeostasis for a particular model organism. To conclude, the reported PG-metrics can really be a strength in high throughput analysis of bacterial cell wall collections.

## Supporting information

S1 TableSimulating the chromatograms.Number of peaks (n), peak positions (p) and width of peaks (w) used to simulate the chromatographic profiles $f(x)=\sum_{i=1}^{n}a_{i}\;{{exp(}^{-}[\frac{\left(x-p_{i}\right)}{w_{i}}]}^{2}$ are summarised below.(DOCX)

S2 Table Optimising the segment length and slack. Variation of the correlation coefficient with segment length at a fixed slack.
(DOCX)

S3 Table Optimising the slack. Variation of the correlation coefficient with slack at a fixed segment length.
(DOCX)

S1 Material Manual for PG-Matrix. (DOCX)

S2 Material PG-Matrix toolbox. (ZIP)
