# Supplementary material for: PG-Metrics: A chemometric-based approach for classifying bacterial peptidoglycan data sets and uncovering their subjacent chemical variability
Source: PLoS One. 2017 Oct 17;12(10):e0186197. doi: 10.1371/journal.pone.0186197 (PMC5645090; doi:10.1371/journal.pone.0186197)
Supplement: S1 Table — Number of peaks (n), peak positions (p) and width of peaks (w) used to simulate the chromatographic profiles f(x)=∑i=1naiexp⁡(−[(x−pi)wi]2 are summarised below. (DOCX) [file pone.0186197.s001.docx]

**S1 Table.** **Simulating the chromatograms.** Number of peaks (n), peak positions (p) and width of peaks (w) used to simulate the chromatographic profiles $f\left( x \right)=\sum_{i=1}^{n} a_{i}{{exp(}^{-}\left[ \frac{(x-p_{i})}{w_{i}} \right]}^{2}$ are summarised below

| **Sample** | **number of**  **peaks (n)** | **Peak position**  **p** | **Amplitude**  **a** | **Width**  **w** |
| --- | --- | --- | --- | --- |
| **1** | **12** | 50 100 170 230 350 400 460 520 590  670 780 900 | 0.30 0.50 0.80  1.00 0.20 0.70  0.30 0.70 0.20  0.65 0.75 0.10 | 3.0 2.0 3.5 4.0 5.2 4.3 6.0 3.0 3.0 2.0 3.5 3.0 |
| **2** | **12** | 60 110 180 240 360 410 470 530 600  680 790 910 | 0.30 0.80 0.20  1.00 0.70 0.50  0.30 0.70 0.20  0.75 0.65 0.10 | 3.0 2.0 3.5 4.0 5.2 4.3 6.0 3.0 3.0 2.0 3.5 3.0 |
| **3** | **12** | 51 102 175 235 353 397 463 523 585 665 785 905 | 0.30 0.80 0.20  1.00 0.70 0.50  0.30 0.70 0.20  0.75 0.65 0.10 | 3.0 2.0 3.5 4.0 5.2 4.3 6.0 3.0 3.0 2.0 3.5 3.0 |
| **4** | **12** | 48 98 171 233 351 403 460 519 585 658 787 902 | 0.45 0.65 0.35  0.90 0.85 0.35  0.30 0.64 0.35  0.66 0.71 0.17 | 3.0 2.0 3.5 4.0 5.2 4.3 6.0 3.0 3.0 2.0 3.5 3.0 |
| **5** | **12** | 53 97 168 231 348 401 457 516 588 661 789 898 | 0.42 0.64 0.32  0.88 0.87 0.3  0.34 0.65 0.38  0.63 0.77 0.19 | 3.0 2.0 3.5 4.0 5.2 4.3 6.0 3.0 3.0 2.0 3.5 3.0 |
| **6** | **12** | 50 100 170 227 350 401 460 520 592 660 792 902 | 0.48 0.60 0.35  0.80 0.89 0.35  0.44 0.61 0.43  0.67 0.76 0.21 | 3.0 2.0 3.5 4.0 5.2 4.3 6.0 3.0 3.0 2.0 3.5 3.0 |
| **7** | **12** | 53 102 174 232 353 403 463 518 596 657 797 903 | 0.53 0.63 0.37  0.82 0.85 0.31  0.47 0.67 0.47  0.62 0.71 0.24 | 3.0 2.0 3.5 4.0 5.2 4.3 6.0 3.0 3.0 2.0 3.5 3.0 |
| **8** | **12** | 51 104 172 235 347 405 468 521 590 660 790 903 | 0.57 0.73 0.31  0.89 0.81 0.25  0.4 0.72 0.48  0.63 0.73 0.18 | 3.0 2.0 3.5 4.0 5.2 4.3 6.0 3.0 3.0 2.0 3.5 3.0 |
| **9** | **12** | 55 107 177 230 349 410 477 528 587 667 798 907 | 0.45 0.68 0.37  0.79 0.89 0.27  0.47 0.75 0.58  0.73 0.83 0.20 | 3.0 2.0 3.5 4.0 5.2 4.3 6.0 3.0 3.0 2.0 3.5 3.0 |
| **10** | **12** | 50 112 167 240 359 420 487 538 598 657 798 912 | 0.55 0.78 0.43  0.89 0.79 0.37  0.57 0.70 0.68  0.83 0.93 0.23 | 3.0 2.0 3.5 4.0 5.2 4.3 6.0 3.0 3.0 2.0 3.5 3.0 |
| **11** | **12** | 55 117 165 245 349 425 497 542 590 650 795 905 | 0.65 0.88 0.48  0.75 0.82 0.38  0.56 0.60 0.88  0.70 0.93 0.20 | 3.0 2.0 3.5 4.0 5.2 4.3 6.0 3.0 3.0 2.0 3.5 3.0 |
| **12** | **12** | 63 115 170 255 365 425 495 552 590 670 800 890 | 0.67 0.88 0.39  0.75 0.32 0.58  0.40 0.65 1.10  0.70 0.90 0.28 | 3.0 2.0 3.5 4.0 5.2 4.3 6.0 3.0 3.0 2.0 3.5 3.0 |
| **13** | **11** | 63 170 255 365 425 495 552 590 670 800 890 | 0.67 0.39 0.75  0.32 0.58 0.40  0.65 1.10 0.70  0.90 0.28 | 3.0 3.5 4.0 5.2 4.3 6.0 3.0 3.0 2.0 3.5 3.0 |
| **14** | **11** | 55 113 173 251 360 421 495 548 594 800 890 | 0.48 0.60 0.35  0.80 0.89 0.45  0.54 0.51 0.45  0.66 0.25 | 3.0 2.0 3.5 4.0 5.2 4.3 6.0 3.0 3.0 3.5 3.0 |
| **15** | **11** | 55 110 167 245 355 425 535 598 660 800 905 | 0.48 0.60 0.35  0.80 0.89 0.45  0.5400 0.51 0.45  0.66 0.25 | 3.0 2.0 3.5 4.0 5.2 4.3 3.0 3.0 2.0 3.5 3.0 |
| **16** | **11** | 112 167 240 359 400 467 525 598 657 798 912 | 0.78 0.39 0.65  0.42 0.58 0.40  0.65 0.80 0.70  0.90 0.38 | 2.0 3.5 4.0 5.2 4.3 6.0 3.0 3.0 2.0 3.5 3.0 |
| **17** | **11** | 51 102 175 235 353 397 463 523 585 665 785 | 0.30 0.80 0.20  1.00 0.70 0.50  0.30 0.70 0.20  0.75 0.65 | 3.0 2.0 3.5 4.0 5.2 4.3 6.0 3.0 3.0 2.0 3.5 3.0 |
| **18** | **12** | 50 100 170 230 350 400 460 520 590 670 780 900 | 0.30 0.80 0.20  1.00 0.70 0.50  0.30 0.70 0.20  0.75 0.65 0.10 | 3.0 2.0 3.5 4.0 5.2 4.3 6.0 3.0 3.0 2.0 3.5 3.0 |
| **19** | **12** | 45 105 165 225 345 385 455 510 585 675 785 904 | 0.31 0.85 0.23  0.95 0.68 0.54  0.32 0.72 0.21  0.80 0.65 0.30 | 3.0 2.0 3.5 4.0 5.2 4.3 6.0 3.0 3.0 2.0 3.5 3.0 |
| **20** | **12** | 48 95 167 228 350 395 465 520 580 685 795 901 | 0.41 0.80 0.21  0.85 0.78 0.50  0.38 0.54 0.71  0.80 0.68 0.32 | 3.0 2.0 3.5 4.0 5.2 4.3 6.0 3.0 3.0 2.0 3.5 3.0 |
| **21** | **12** | 48 100 170 220 355 405 475 522 585 695 793 907 | 0.71 0.45 0.31  0.45 0.85 0.350  0.40 0.68 0.61  0.70 0.78 0.38 | 3.0 2.0 3.5 4.0 5.2 4.3 6.0 3.0 3.0 2.0 3.5 3.0 |
|  |  |  |  |  |
