# Supplementary material for: PG-Metrics: A chemometric-based approach for classifying bacterial peptidoglycan data sets and uncovering their subjacent chemical variability
Source: PLoS One. 2017 Oct 17;12(10):e0186197. doi: 10.1371/journal.pone.0186197 (PMC5645090; doi:10.1371/journal.pone.0186197)
Supplement: S2 Table — Variation of the correlation coefficient with segment length at a fixed slack. (DOCX) [file pone.0186197.s002.docx]

**S2 Table. Optimising the segment length.** Variation of the correlation coefficient with segment length at a fixed slack

| **Segment length** | **Slack** | **Correlation coefficient (ρ)** |
| --- | --- | --- |
| 100 | 20 | 0.776 |
| 90 | 20 | 0.758 |
| 80 | 20 | 0.789 |
| 70 | 20 | 0.687 |
| 60 | 20 | 0.804 |
| 50 | 20 | 0.859 |
| 40 | 20 | 0.881 |
| 35 | 20 | 0.884 |
| 30 | 20 | 0.879 |
