# Supplementary material for: PG-Metrics: A chemometric-based approach for classifying bacterial peptidoglycan data sets and uncovering their subjacent chemical variability
Source: PLoS One. 2017 Oct 17;12(10):e0186197. doi: 10.1371/journal.pone.0186197 (PMC5645090; doi:10.1371/journal.pone.0186197)
Supplement: S3 Table — Variation of the correlation coefficient with slack at a fixed segment length. (DOCX) [file pone.0186197.s003.docx]

**S3 Table. Optimising the slack.** Variation of the correlation coefficient with slack at a fixed segment length

| **Slack** | **Segment length** | **Correlation coefficient (ρ)** |
| --- | --- | --- |
| 1 | 35 | 0.629 |
| 5 | 35 | 0.790 |
| 10 | 35 | 0.873 |
| 15 | 35 | 0.883 |
| 20 | 35 | 0.884 |
| 25 | 35 | 0.884 |
| 30 | 35 | 0.884 |
