# Supplementary material for: PG-Metrics: A chemometric-based approach for classifying bacterial peptidoglycan data sets and uncovering their subjacent chemical variability
Source: PLoS One. 2017 Oct 17;12(10):e0186197. doi: 10.1371/journal.pone.0186197 (PMC5645090; doi:10.1371/journal.pone.0186197)
Supplement: S1 Material — (DOCX) [file pone.0186197.s004.docx]

**S1 Material. Manual for Peptidoglycan-Matrix**

1. Unzip the Peptidoglycan Matrix folder by write click and extract all


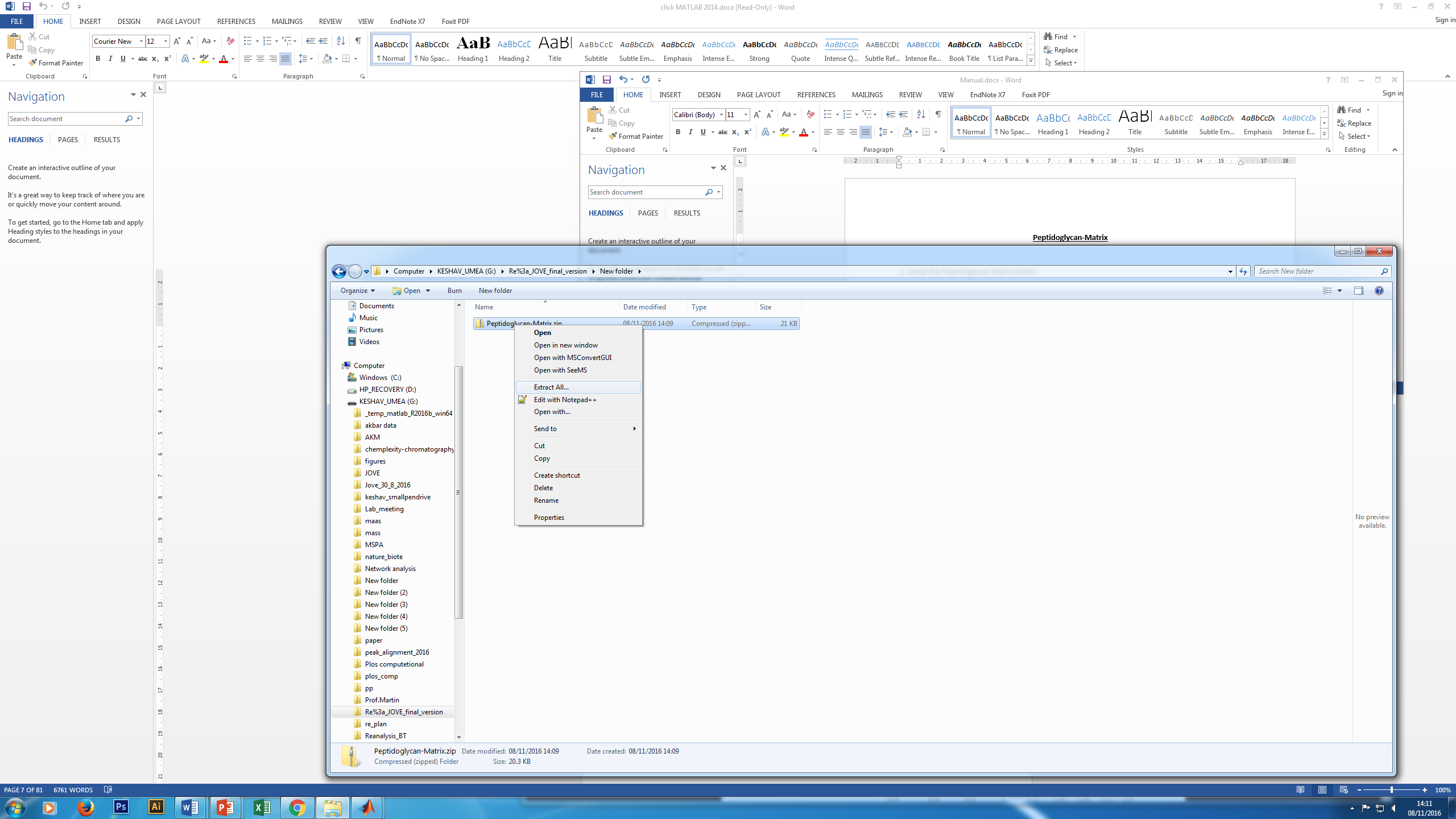


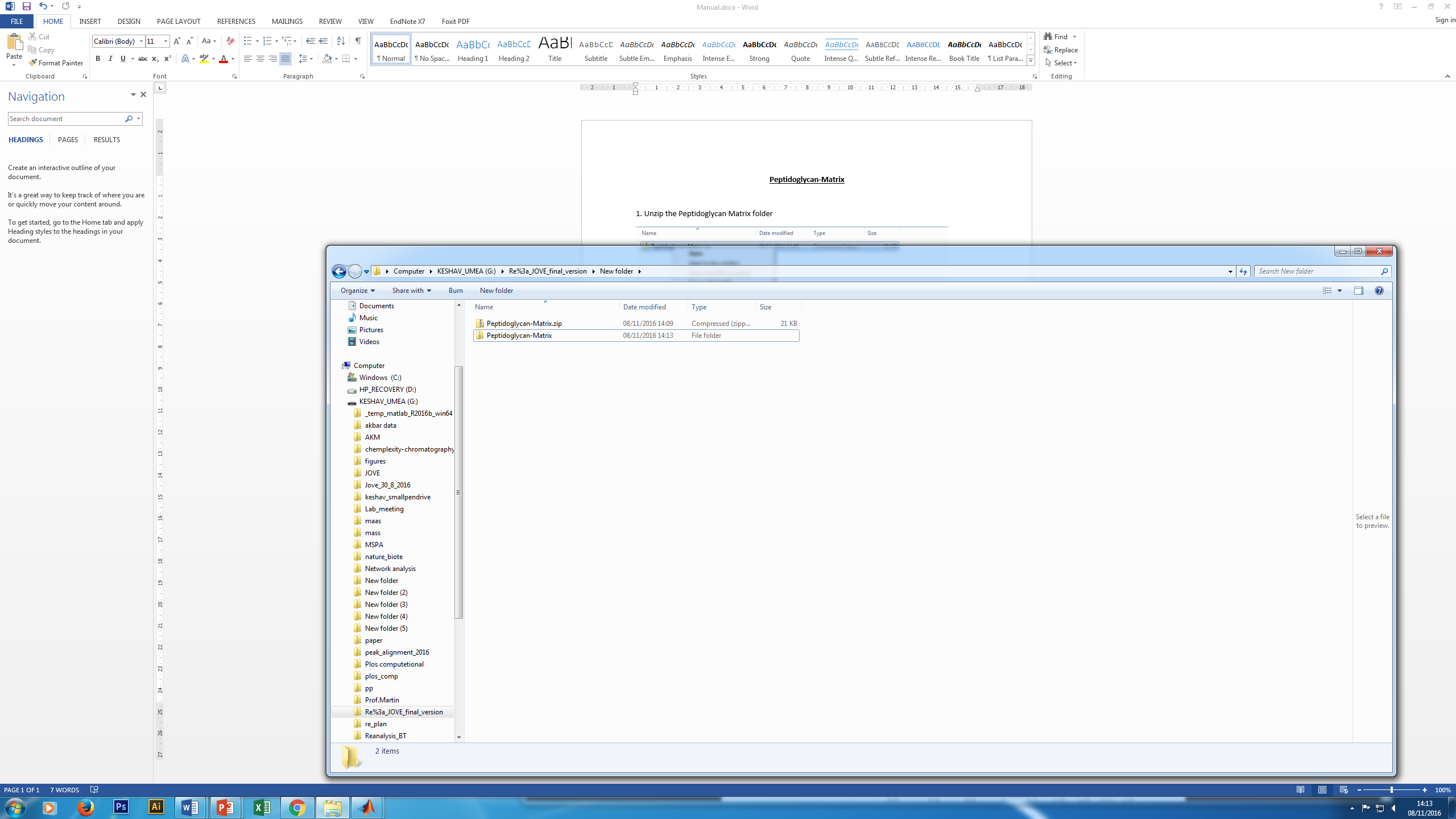


2. Double click MATLAB


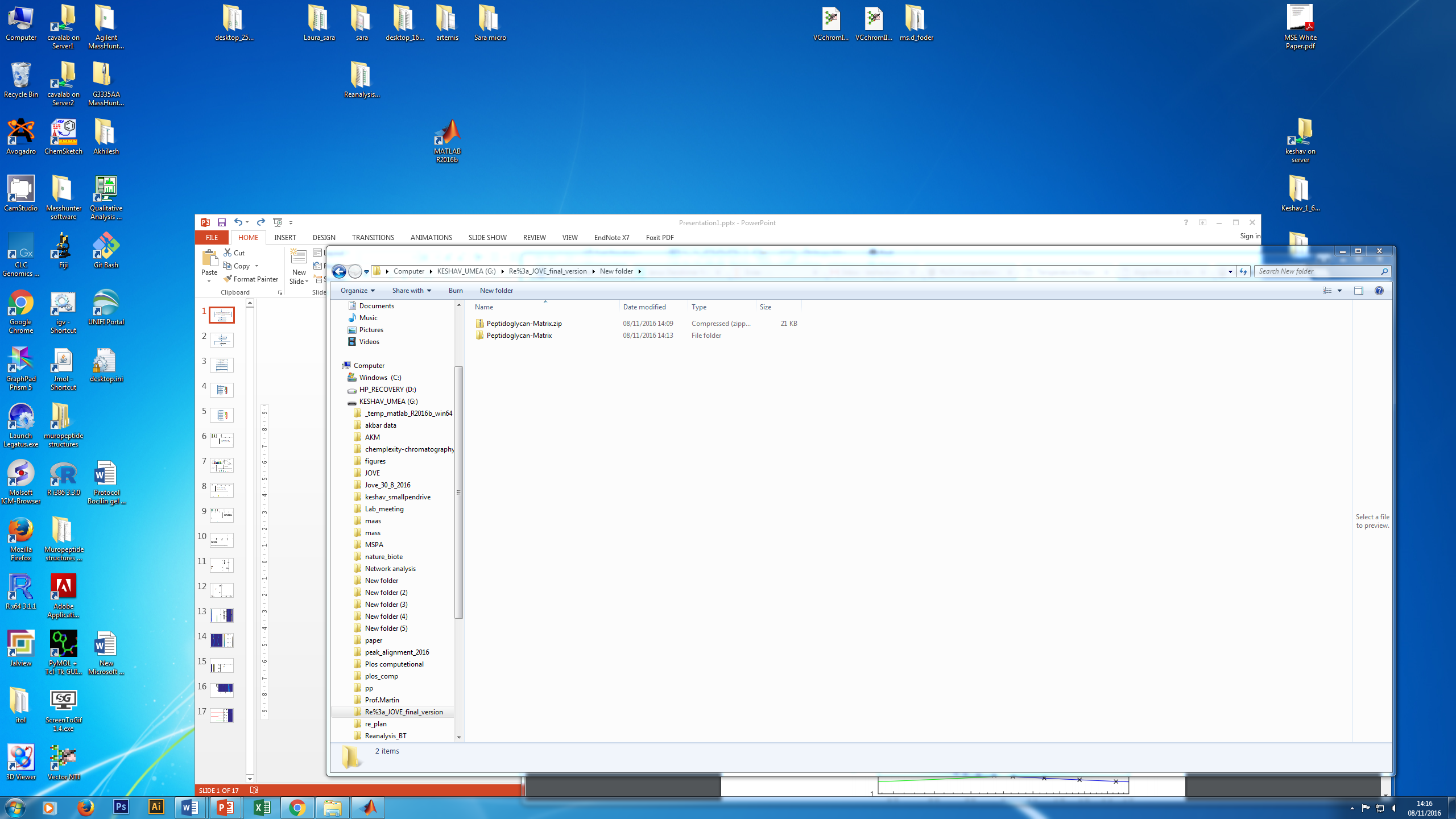


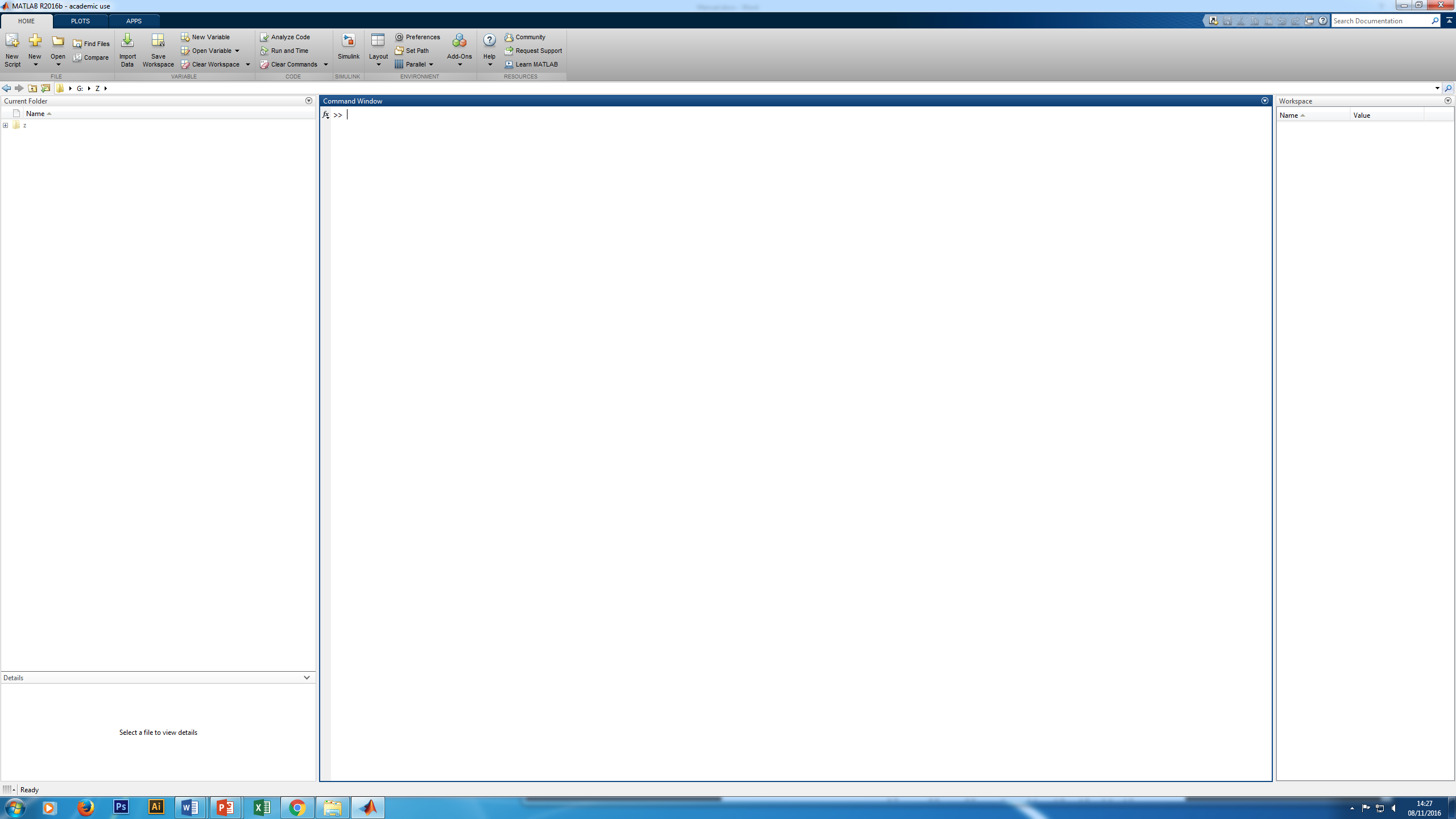


3. Copy the Peptidoglycan Matrix folder


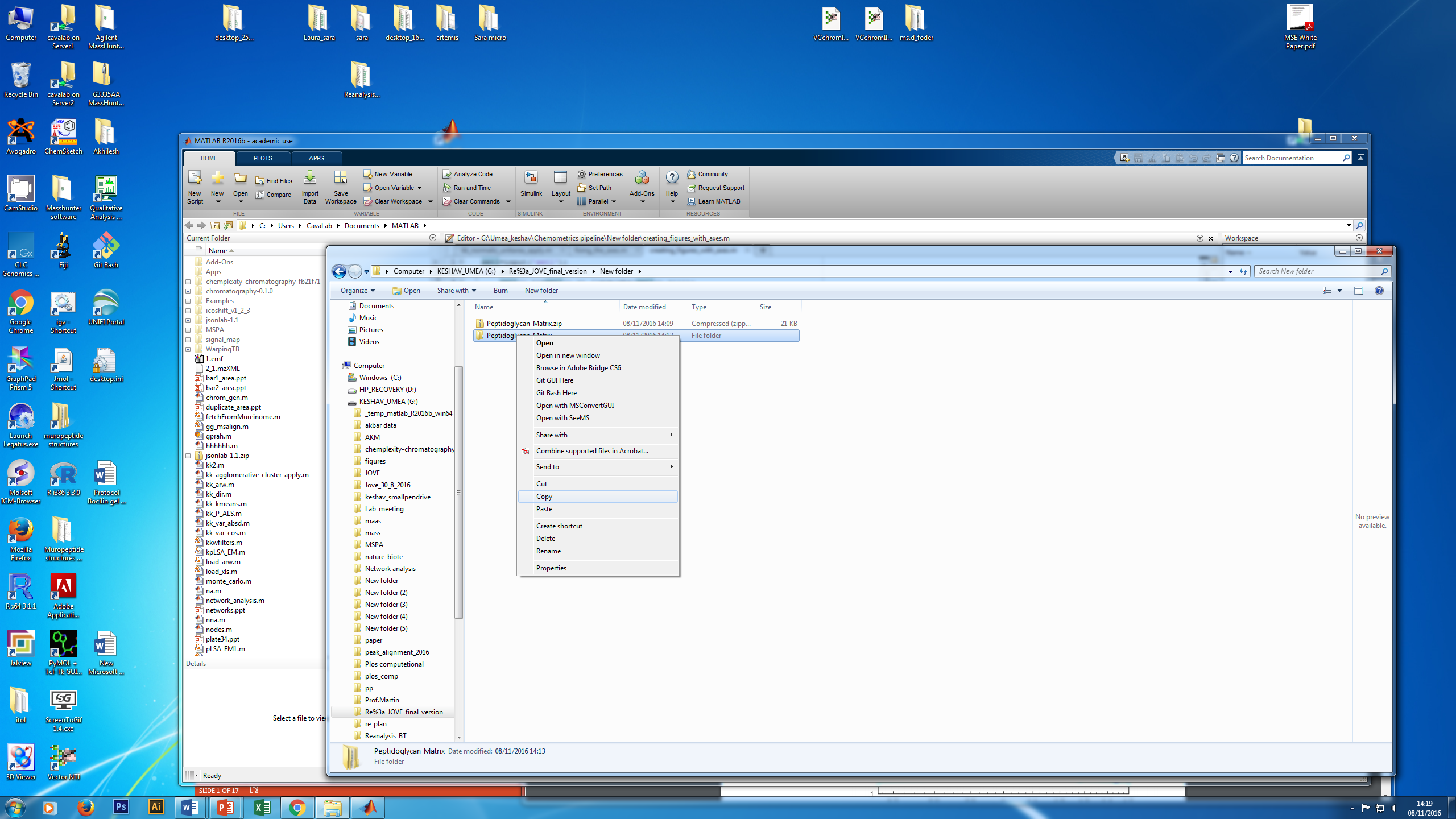


4. Paste the Peptidoglycan Matrix folder


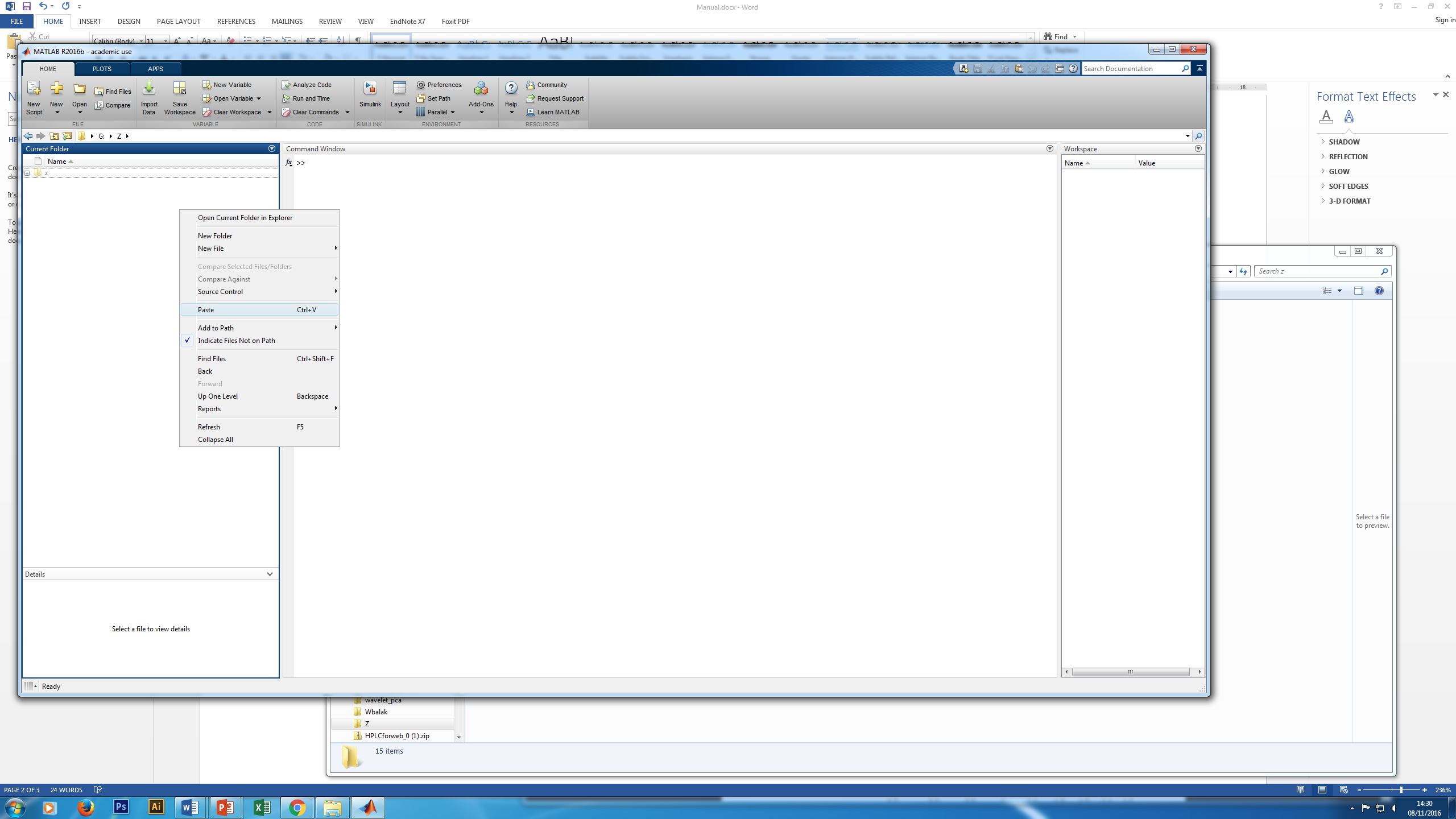


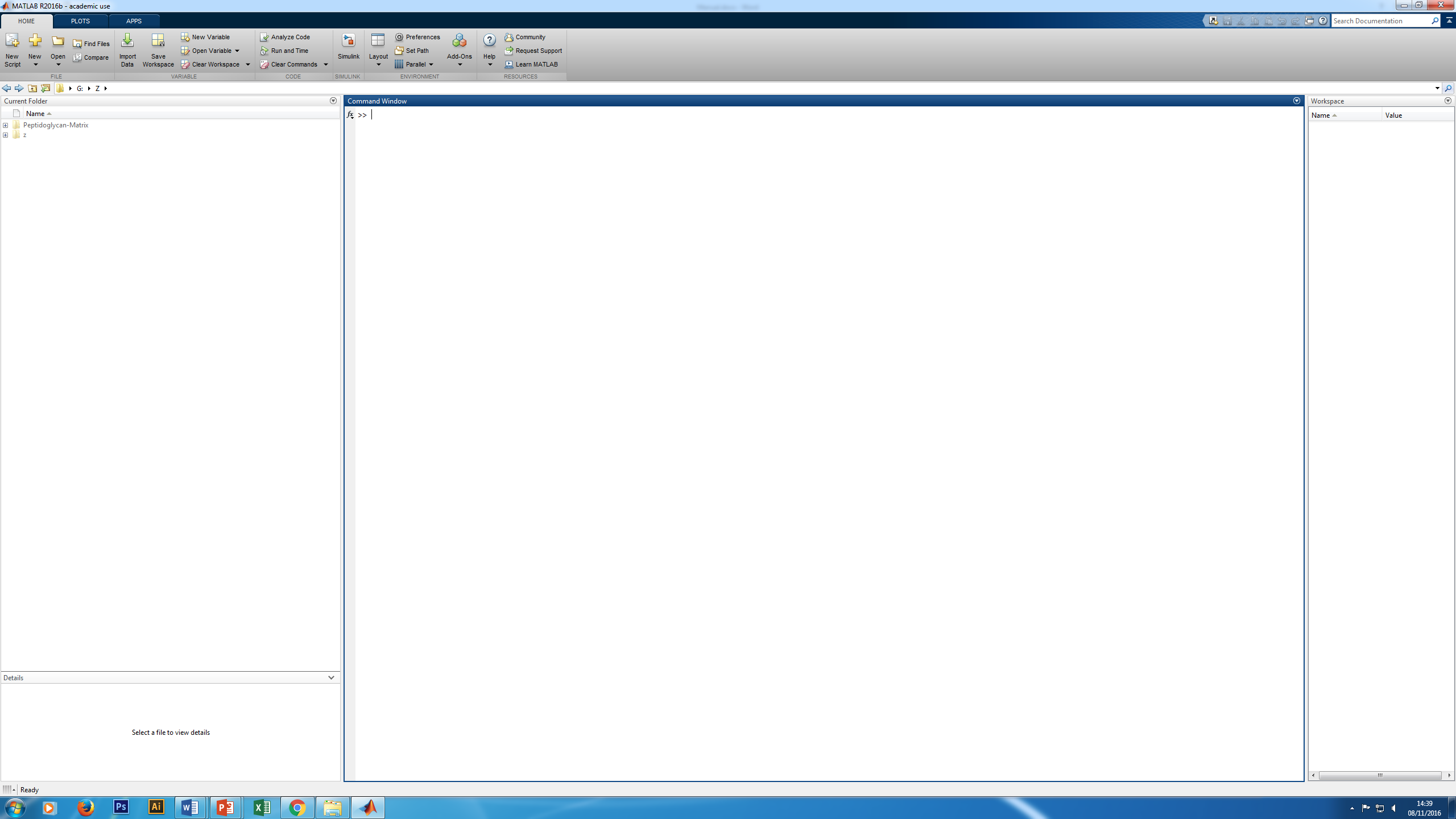


5. Double click the Peptidoglycan Folder


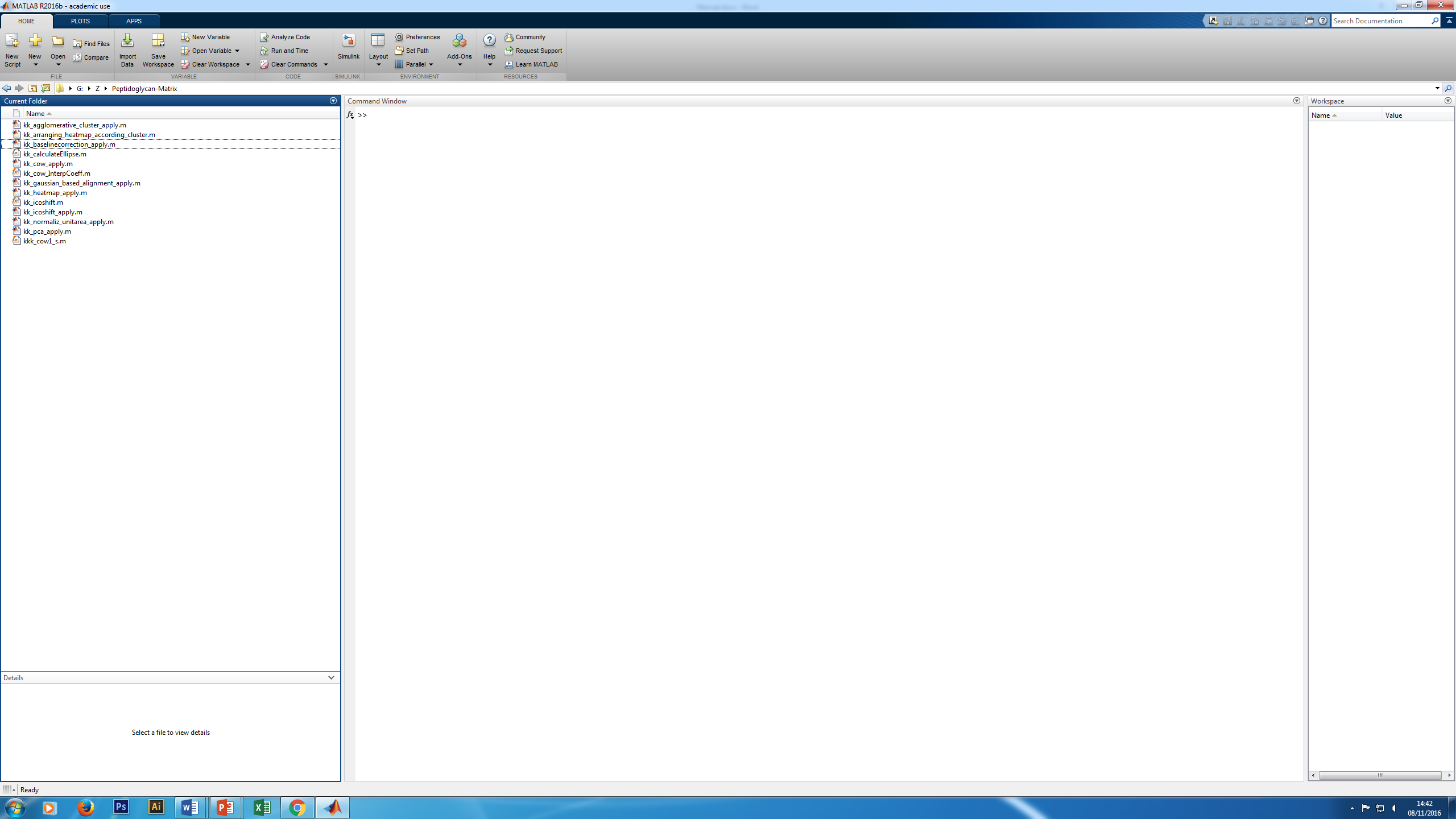


6. Import the data


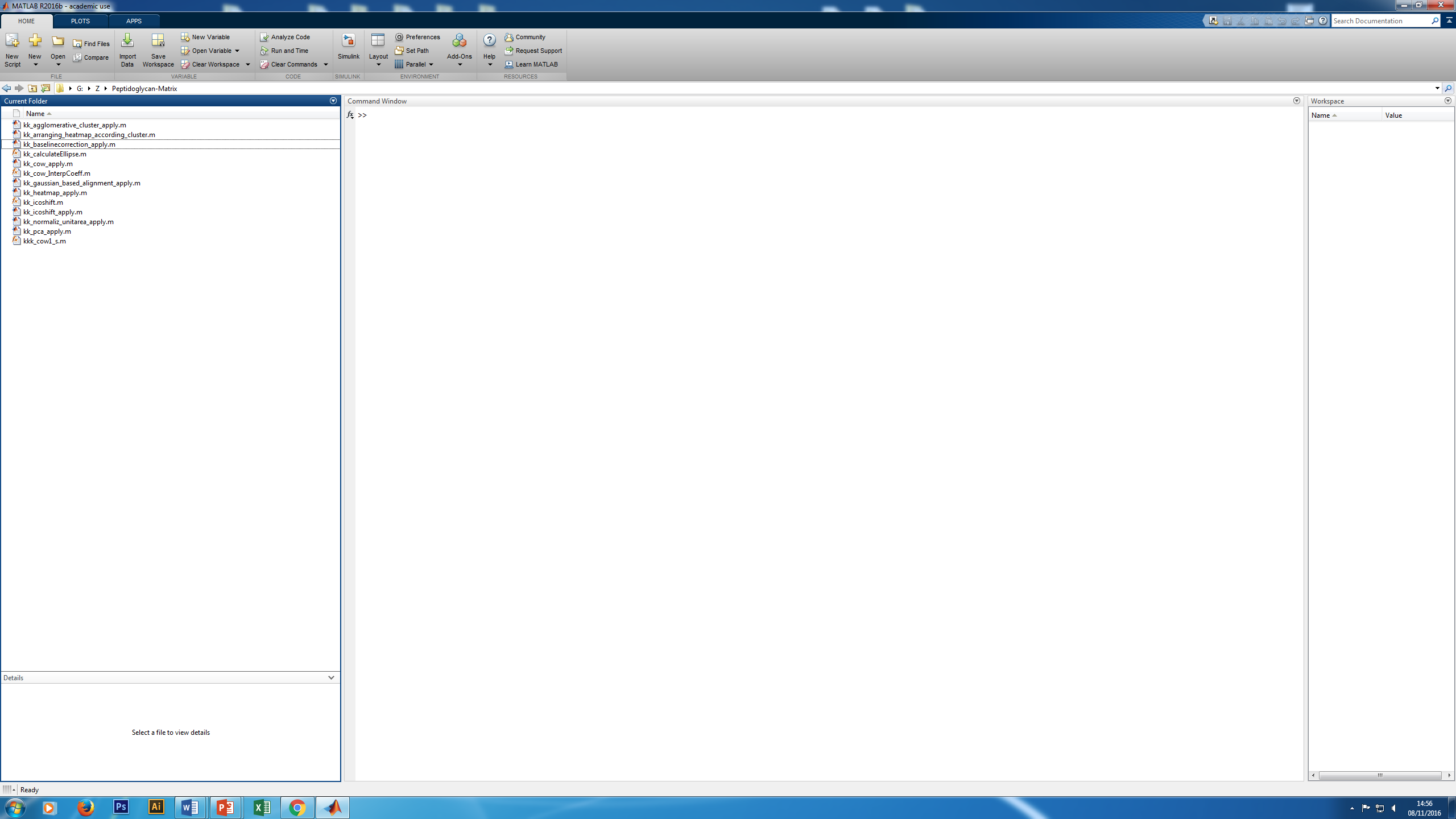


Click the Import Data

>> Go to the desired folder and select the excel file containing the data


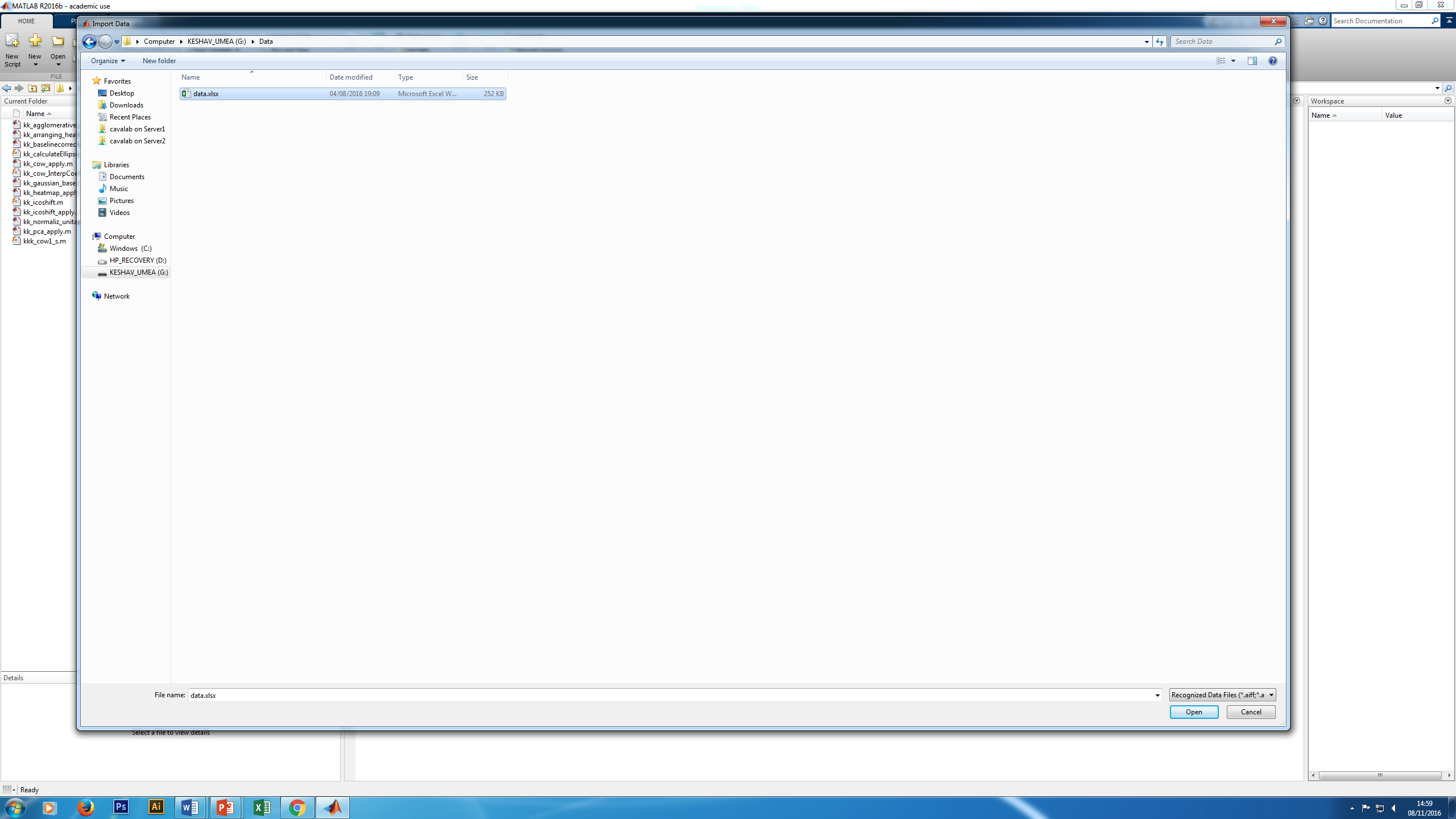


Click Open


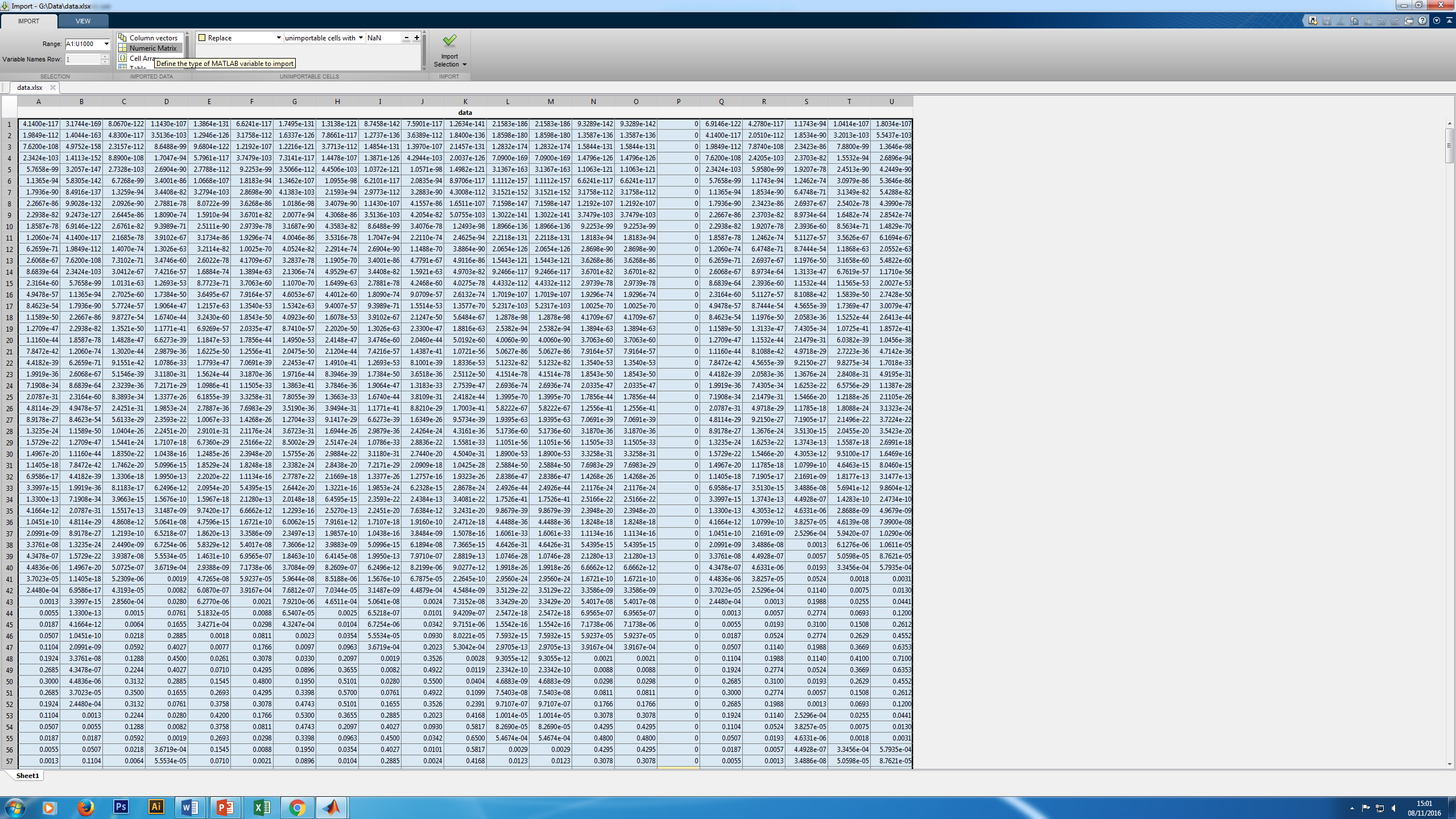


Select Matrix


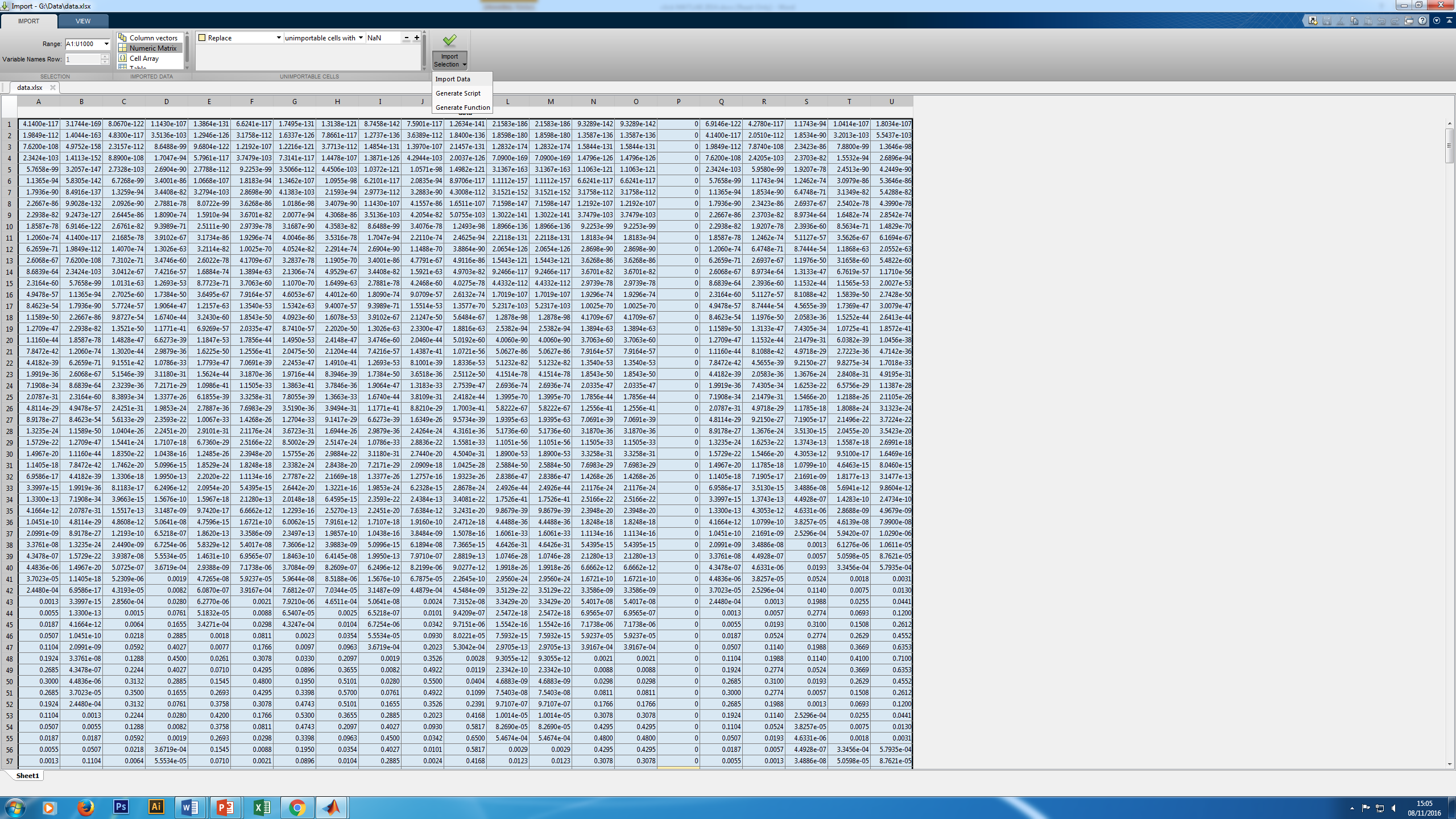


Click Import selection

Then click Import Data

Import data

‘

Imported Data can be seen on the workspace (right-side)


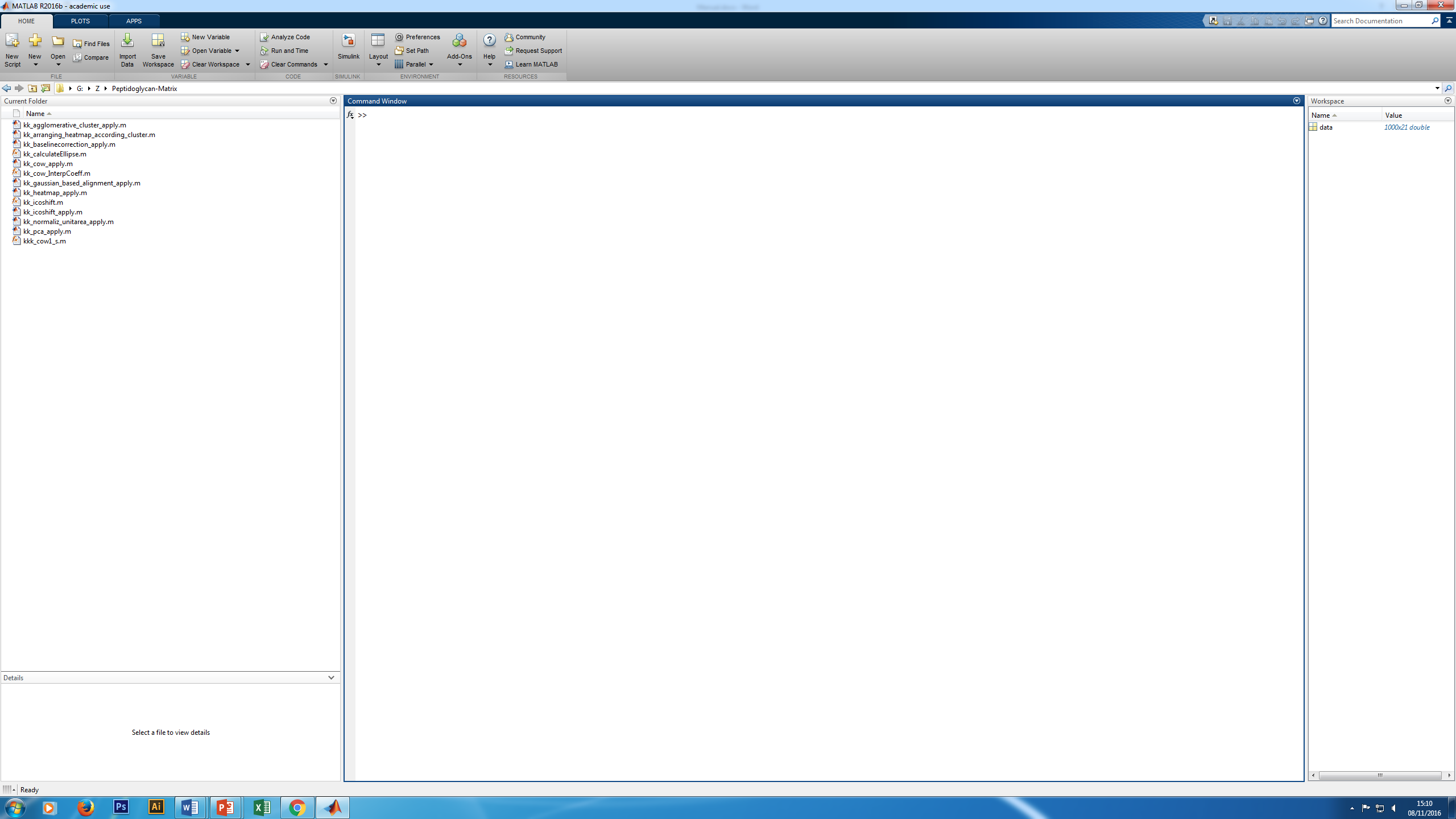


Imported Data

‘

Variable can be rename by right clicking


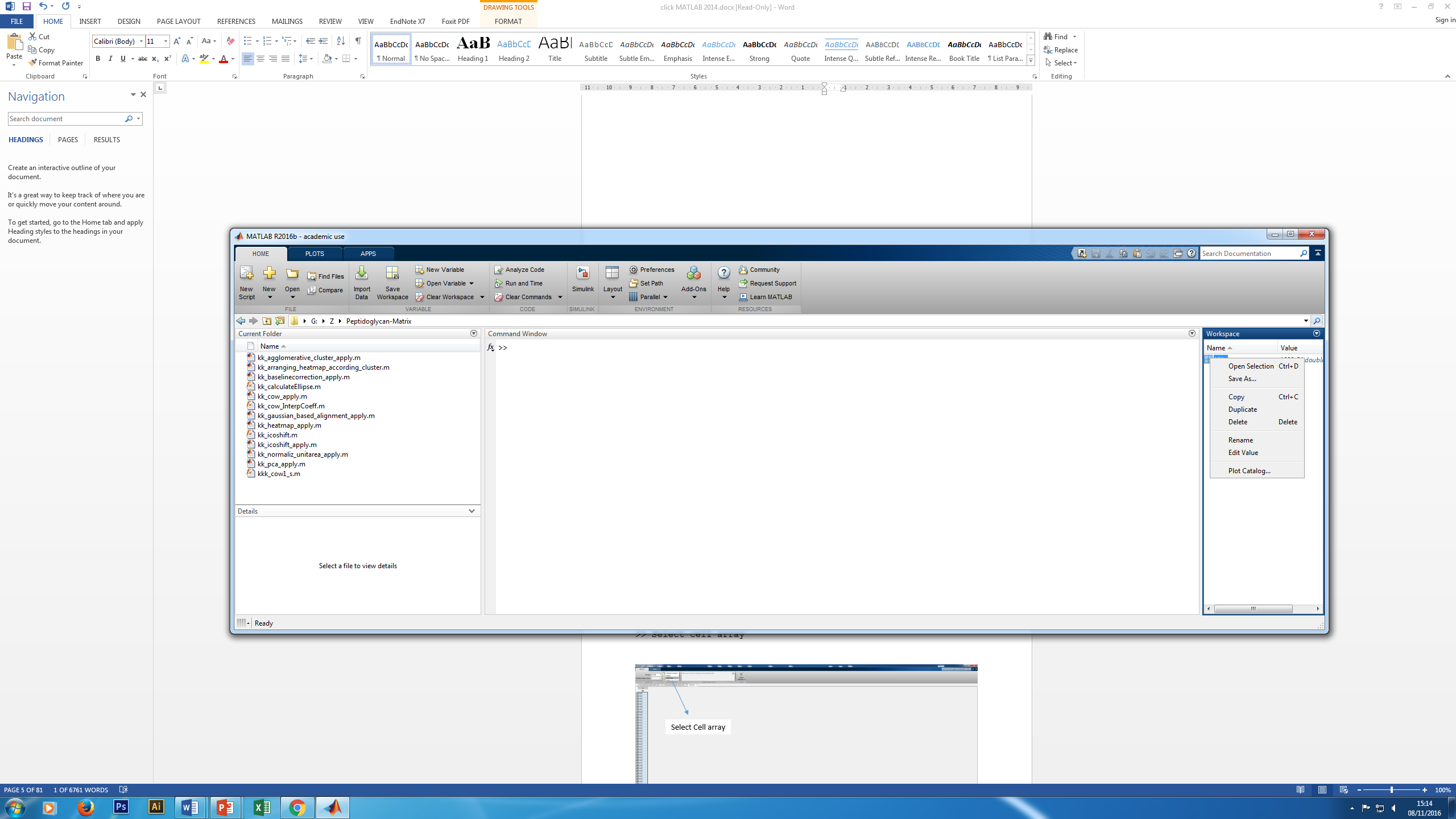


Renamed as XX

‘

7. Importing the Labels

Click import data ---------------------> Go to the folder containing the excel file --------------> click open -----------> select cell array-----------------> Import Data


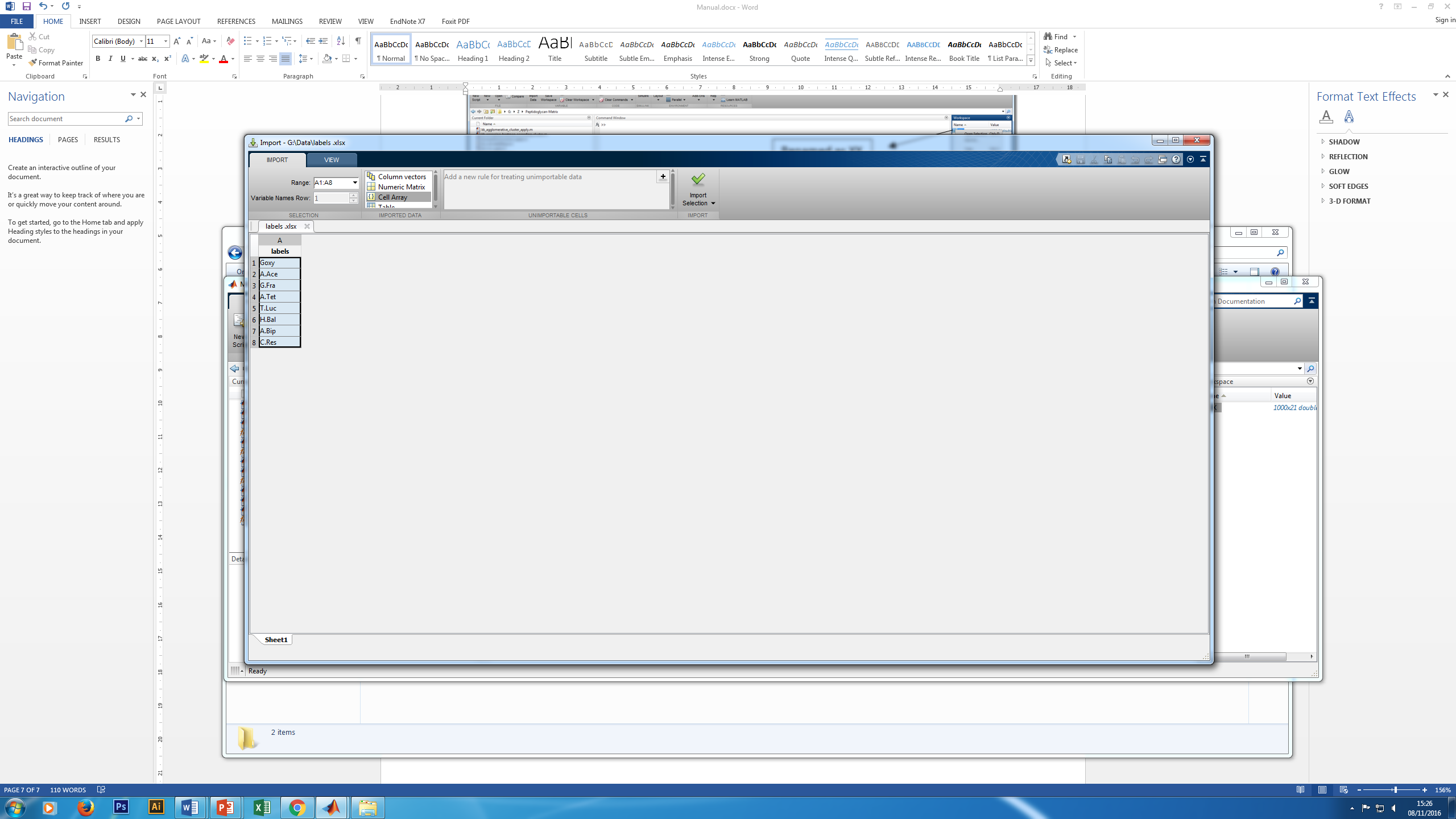


Select cell array

‘


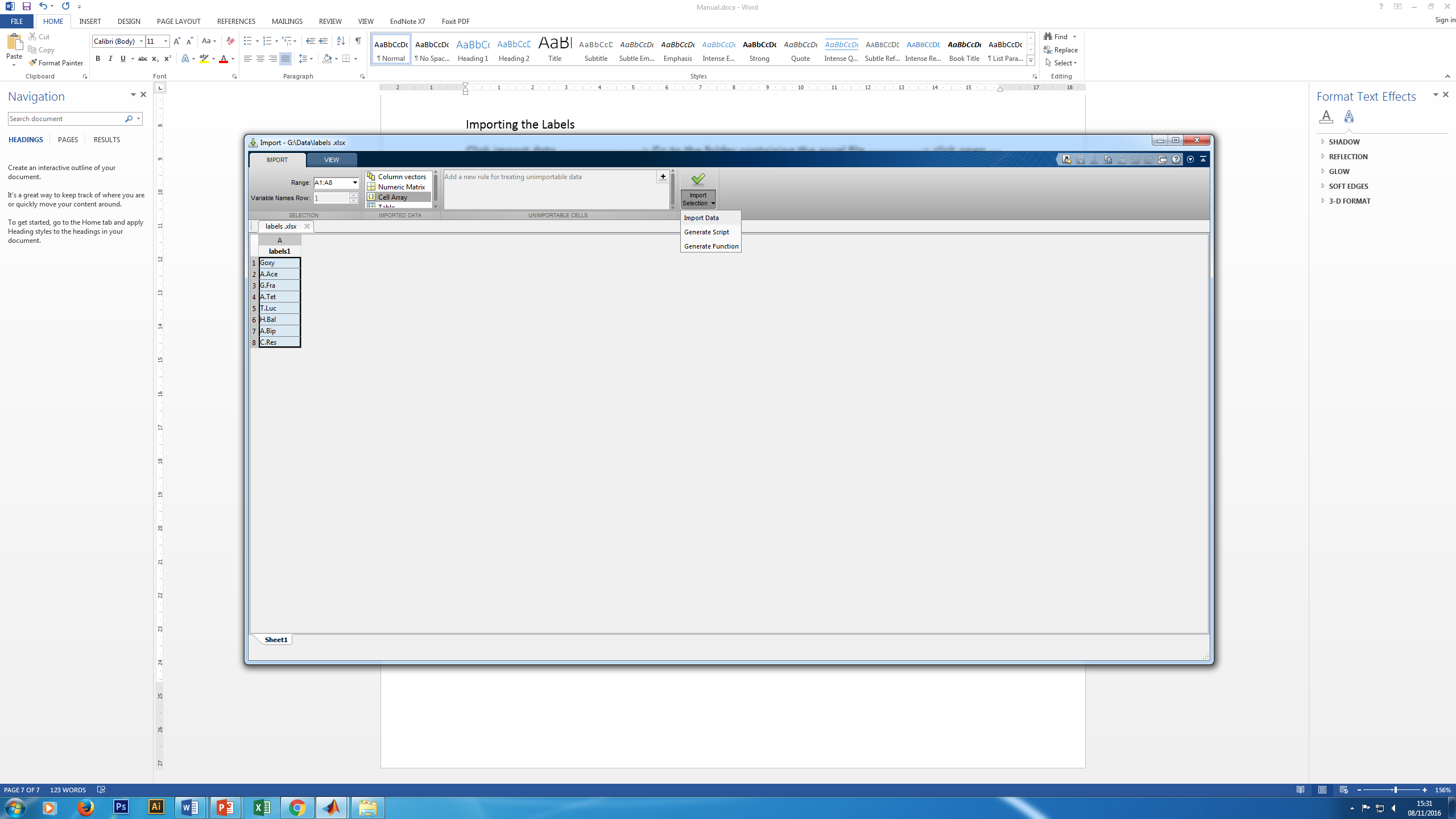


Click Import selection

Then click Import Data

Import data

‘


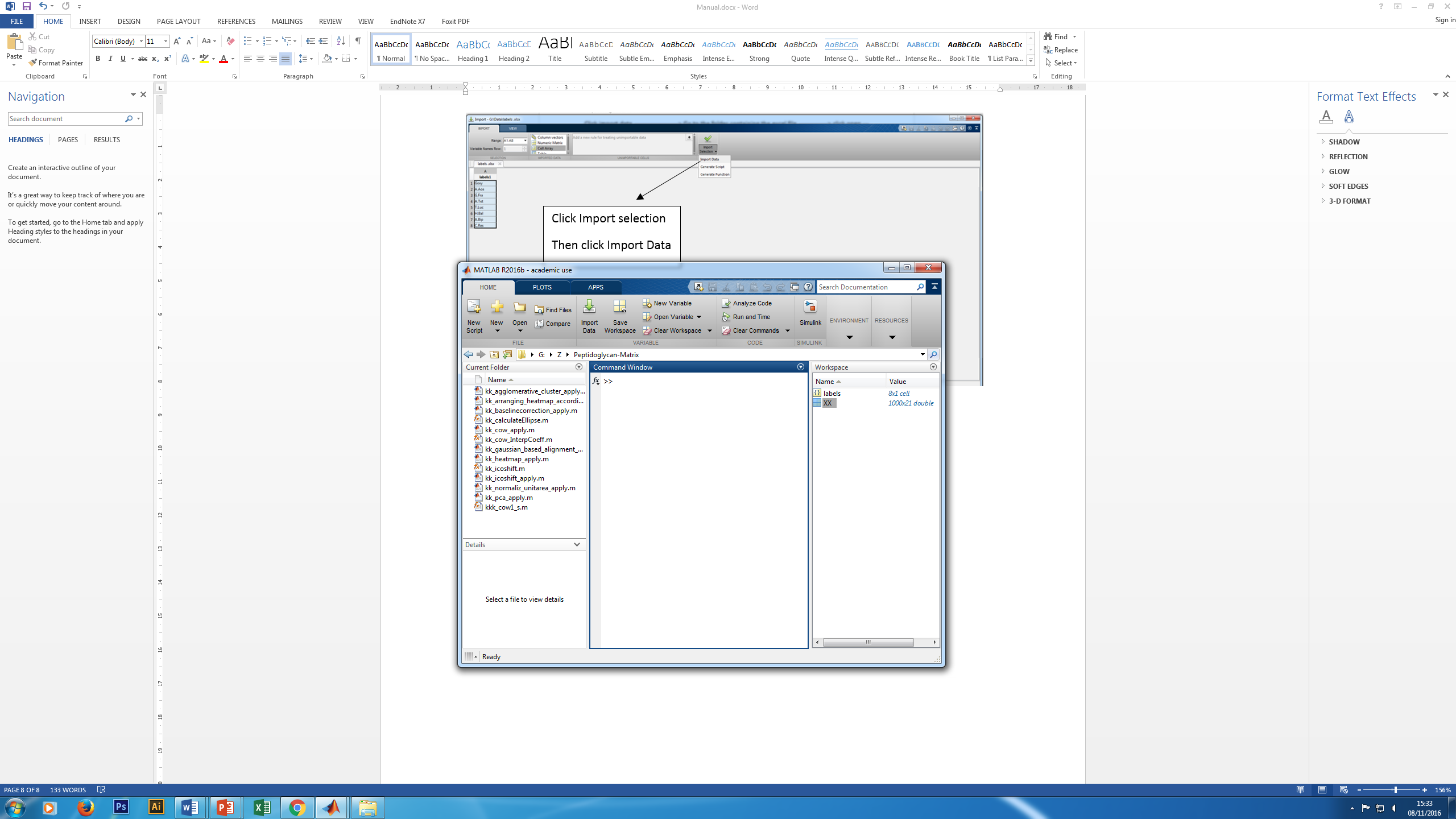


Imported Labels

‘

......................................................................................................................................................

8. Baseline correction

Do the following in the MATLAB command window

Baseline correction


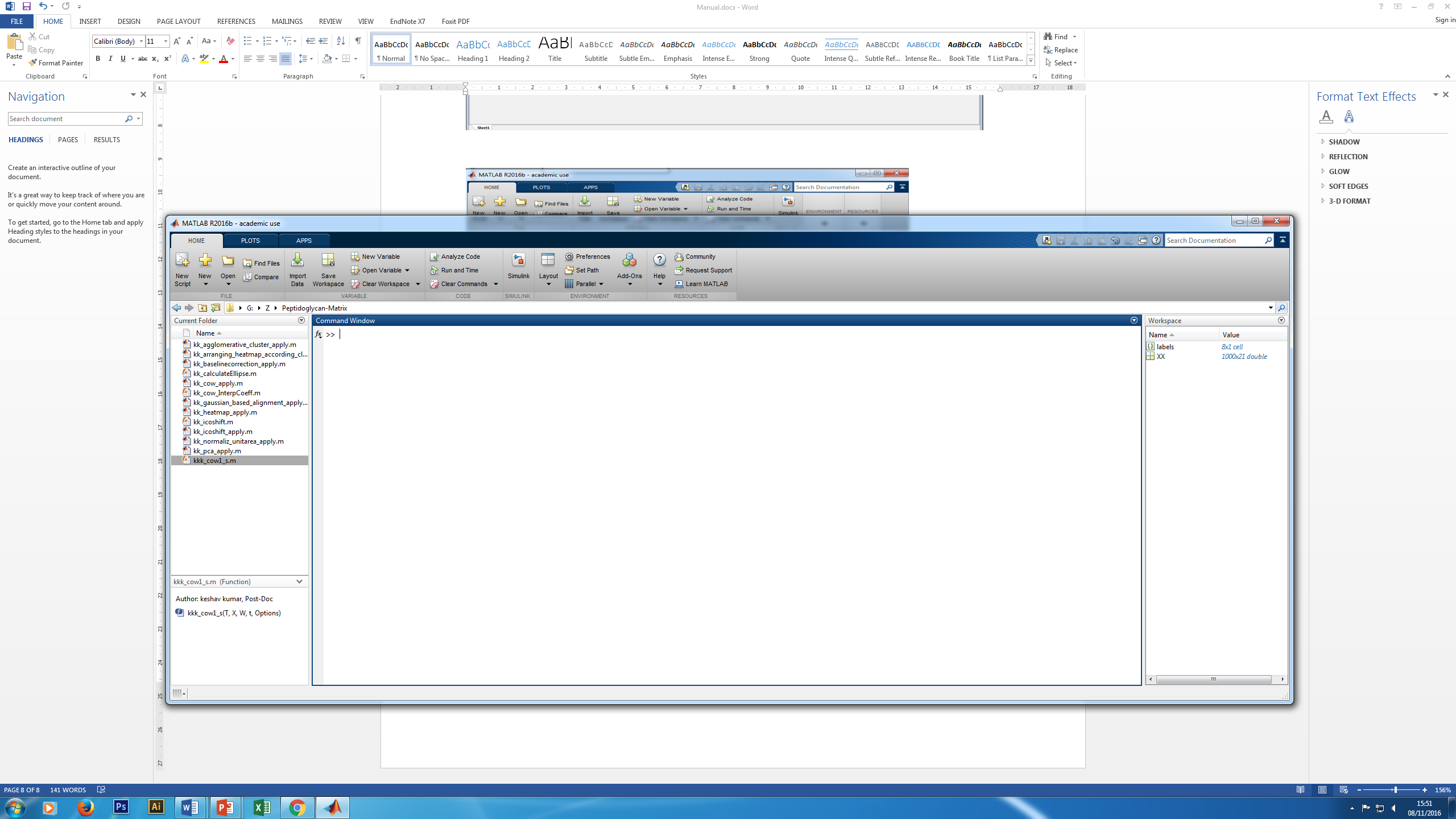


Type kk_baselinecorrection_apply and enter

‘


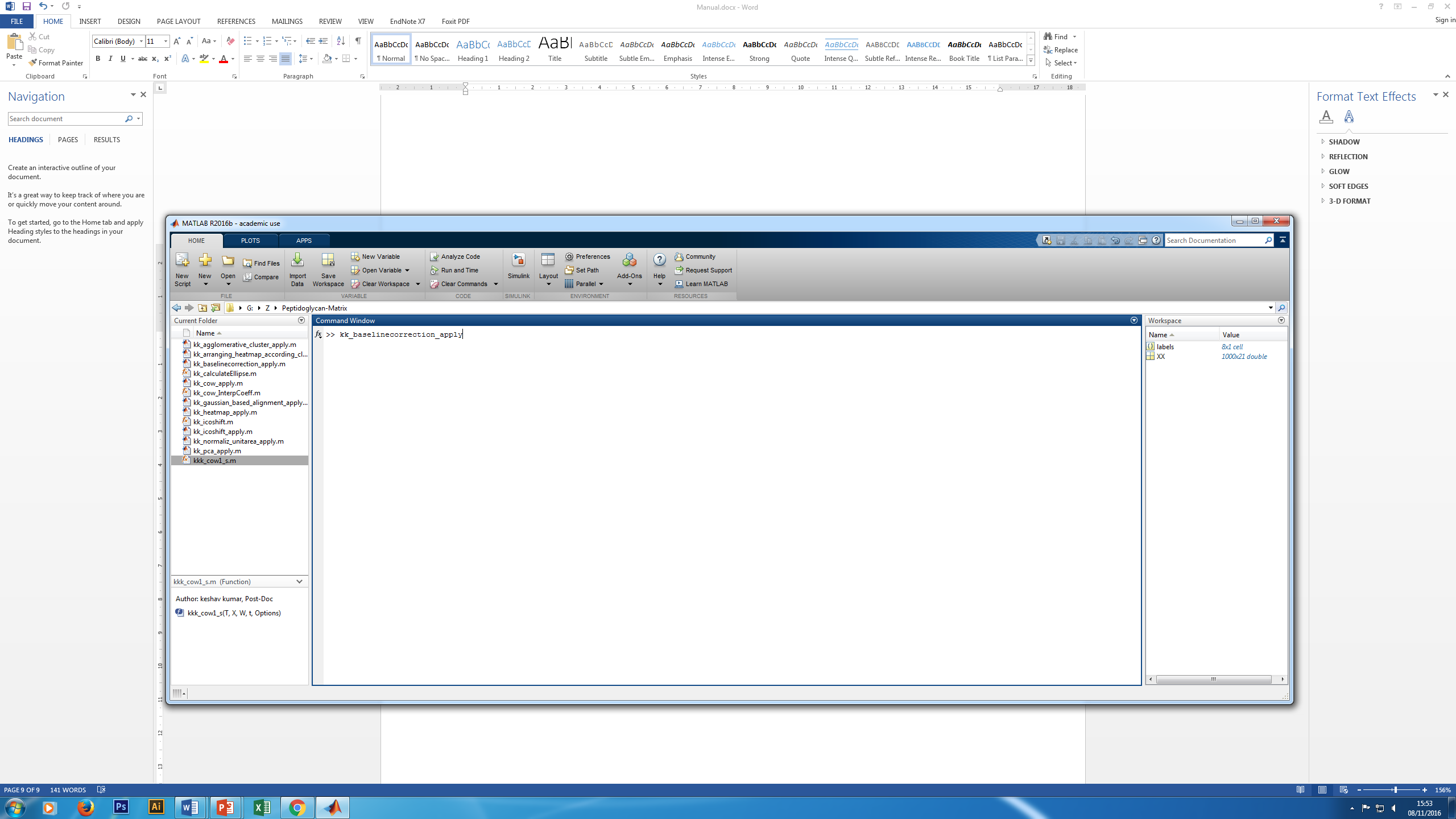


Provide the following information in sequence

data =......................................................................... (provide the data to be baseline corrected)

windowsize = ............................................................ (Specify the windowsize)

stepsize = .................................................................. (Specify the stepsize)

quantile value = .........................................................(Specify the quantile value)

The baseline corrected data will be in the matrix yy.

---------------------------------------------------------------------------------------------------------------

9. Correlation optimised warping (COW) of the data sets

Do the following in the MATLAB command window

>> kk_cow_apply

>> Target = .......................................................................... (Specify the target chromatogram)

>> samples_to_be_aligned = ................................................ (Specify the samples to be aligned)

>> segment_length = ............................................................. (Specify the segment length)

>> slack = ............................................................................. (Specify the slack)

Y_aligned matrix in the MATLAB contains the aligned chromatograms

------------------------------------------------------------------------------------------------------------

10. icoshift analysis on the data sets

Do the following in the MATLAB command window

>> kk_icoshift_apply

>> Target = ........................................................................... (Specify the target chromatogram)

>> samples_to_be_aligned= ................................................ (Specify the samples to be aligned)

>> interval= .......................................................................... (Specify the interval)

Mention the interval, write 'complete' for entire chromatogram, for range write the initial and final value in the following format, [initial_value final_value, intial_value final_value, .....] (i.e. [200 300, 500 600])

Y_aligned matrix in the MATLAB contains the aligned chromatograms

----------------------------------------------------------------------------------------------------------------

11. msalign analysis on the data sets

Do the following in the MATLAB command window

>> kk_msalign_apply

>> data to be aligned = .................................. (Specify the chromatograms need to be aligned)

>> The reference peak positions= ................... (Specify the reference peak position)

>> gaussian_width = ........................................ (Specify the width of reference peaks)

>> range for shifting the time points = ............. [Specify the range for shifting the chromatograms (in the format [-left +right] e.g. [-10 10] or [-37 48])]

----------------------------------------------------------------------------------------------------------------

12. Principal component analysis (PCA)

Do the following in MATLAB command window

>> kk_pca_apply

>> data= .................................................................................... [specify the data]

>> factor = ................................................................................. [specify the number of factor]

>> sample_labels=...................................................................... [specify the labels]

----------------------------------------------------------------------------------------------------------------

13. cluster analysis

Do the following in MATLAB command window

>> kk_agglomerative_cluster_apply

>> choice of linkage= ............................................ [Specify the choice of linkage e.g. ‘ward’]

choose method for calculating the distance= .......... [Specify the method for calculating the distance e.g. ‘euclidean’]

>> data_set= ........................................................... [specify the data set]

>> do_you_want_pca= ........................................... [specify the choice with 1 for yes and 0 for no]

If yes for PCA

>> factor = ............................................................... [specify the number of factors]

if no for PCA

>> do_you_want_normaliz = ................................. [specify the choice with 1 for yes 0 for n0]

The dendrogam can be seen in the figure window

----------------------------------------------------------------------------------------------------------------

14. Heatmap analysis

Do the following in the MATLAB command window

>> kk_heatmap_apply

>> data= ................................................................... [Specify the data for creating the matrix]

>> do_you_want_to_normaliz = .............................. [Specify the option for yes type ‘1’ and type ‘0’ for no]

>> time = ................................................................. [specify the time axis for making the heatmap]

The heat map can be seen in the figure window.

---------------------------------------------------------------------------------------------------------------

15 Arranging the samples in the heatmap according to dendrogram

Do the following in the MATLAB command window

(i) Perform the cluster analysis with appropriate parameters as discussed above with

>> kk_agglomerative_cluster_apply

(ii) type

>> kk_arranging_heatmap_according_cluster

>> parameter = ........................................................... [write perm ]

>> data = ........................................................................ [provide the data that are used for the cluster analysis]

>> do_you_want_to_normaliz = .............................. [Specify the option for yes type ‘1’ and type ‘0’ for no]

>> time = ................................................................. [specify the time axis for making the heatmap]

The heat map arranged as per the dendrogram can be seen in the figure window.

----------------------------------------------------------------------------------------------------------------

**16. Some useful codes:**

(i) To normalize the data to unit area

Do the following in the MATLAB command window

>> kk_normaliz_unitarea_apply

>> data = ..................................................................... [Specify the data to be normalized]

The normalized data can be seen in the matrix xy

(ii) To normalize the data to maxima

Do the following in the MATLAB command window

>> kk_normaliz_max_apply

>> data = ..................................................................... [Specify the data to be normalized]

The normalized data can be seen in the matrix xy

(iii) To auto scale the data

>> kk_autoscale_apply

>> data = ..................................................................... [Specify the data to be autoscaled]

The auto scaled data can be seen in the matrix xy1.

(iv) To mean centering the data

>> kk_meancentering_apply

>> data = ..................................................................... [Specify the data to be mean_centerd]

The mean centerd data can be seen in the matrix xy.

(v) To median centring the data

>> kk_mediancentering_apply

>> data = .................................................................... [Specify the data to be median_centerd]

The median_centerd data can be seen in the matrix xy.

(vi) To mean center the data with unit variance perform SNV (standard normal variate analysis)

>> kk_snv_apply

>> data = .................................................................... [Specify the data to be median_centerd]

The SNV pre-processed data can be seen in the matrix labelled as xy1.

(vii) To transform the data to log scale

>> kk_logscaling_apply

>> data = .................................................................... [Specify the data to be log_scaled]

The log_scaled data can be seen in the matrix labelled as log_xy.

(viii) To transform the data to log10 scale

>> kk_logscaling_10_apply

>> data = .................................................................... [Specify the data to be log_10_scaled]

The log 10 scaled data can be seen in the matrix labelled as log_10_xy.

(ix) To remove the missing values from the data sets

>> kk_missingvalue_handling_apply

>> Y_miss = ................................................................. [Specify the data containing the missing values]

>> value to be assigned to the missing value= ............ [Specify the value need to be assigned to the missing values]

The data set with no missing values can be seen in the matrix labelled as Y_app.

----------------------------------------------------------------------------------------------------------------

17. To perform the PCA without pre-processing the data set

Do the following in the MATLAB command window

>> kk_pca_without_preprocessing_apply

>> data=................................................................................. [Specify the data to be analysed]

>> factor =................................................................................ [Specify the number of factors]

>> sample_label=........................................................................ [Specify the sample labels]
